# Kinesin-1 transports morphologically distinct intracellular virions during vaccinia infection

**DOI:** 10.1242/jcs.260175

**Published:** 2022-09-30

**Authors:** Amadeus Xu, Angika Basant, Sibylle Schleich, Timothy P. Newsome, Michael Way

**Affiliations:** ^1^ Cellular signalling and cytoskeletal function laboratory, The Francis Crick Institute, 1 Midland Road, London, NW1 1AT, UK; ^2^ London Research Institute, Cancer Research UK, 44 Lincoln's Inn Fields, London, WC2A 3PX, UK; ^3^ Department of Infectious Disease, Imperial College, London W2 1PG, UK

**Keywords:** Vaccinia virus, Kinesin-1, Microtubule transport, *In vitro* motility assays

## Abstract

Intracellular mature viruses (IMVs) are the first and most abundant infectious form of vaccinia virus to assemble during its replication cycle. IMVs can undergo microtubule-based motility, but their directionality and the motor involved in their transport remain unknown. Here, we demonstrate that IMVs, like intracellular enveloped viruses (IEVs), the second form of vaccinia that are wrapped in Golgi-derived membranes, recruit kinesin-1 and undergo anterograde transport. *In vitro* reconstitution of virion transport in infected cell extracts revealed that IMVs and IEVs move toward microtubule plus ends with respective velocities of 0.66 and 0.56 µm/s. Quantitative imaging established that IMVs and IEVs recruit an average of 139 and 320 kinesin-1 motor complexes, respectively. In the absence of kinesin-1, there was a near-complete loss of *in vitro* motility and reduction in the intracellular spread of both types of virions. Our observations demonstrate that kinesin-1 transports two morphologically distinct forms of vaccinia. Reconstitution of vaccinia-based microtubule motility *in vitro* provides a new model to elucidate how motor number and regulation impacts transport of a bona fide kinesin-1 cargo.

## Teaser

**Summary:** The kinesin-1 motor mediates anterograde microtubule-based transport of the intracellular mature and enveloped forms of vaccinia virus in cells and *in vitro*.

## INTRODUCTION

Kinesin-1, the founding member of the kinesin superfamily, anchors and transports a diverse range of cellular cargoes, including vesicles, organelles, protein complexes and ribonucleoproteins towards the plus end of microtubules (Cai et al., 2009; Hirokawa et al., 2009). Kinesin-1 is a heterotetramer consisting of two heavy chains, each of which contains an N-terminal motor domain that is necessary for movement, and two light chains, which play important roles in motor regulation and cargo binding (Bloom et al., 1988; Hackney and Stock, 2000; Kaan et al., 2011; Vale et al., 1985). In humans, the kinesin heavy chain is represented by three different genes that encode closely related protein isoforms, KIF5A, KIF5B and KIF5C. KIF5B appears to be ubiquitously expressed, whereas KIF5A and KIF5C are neuronal specific (Kanai et al., 2000). Each KIF5 heavy-chain homodimer associates near the C-termini of its subunits with the heptad repeats of two copies of one of four light-chain isoforms (KLC1–4) (Miki et al., 2001). Although KLC2 is ubiquitously expressed and KLC1 is found in most cell types, the other isoforms are tissue specific (Junco et al., 2001; Rahman et al., 1998). Despite the importance of kinesin-1 in the transport of many cellular cargoes, we lack a thorough understanding of kinesin-1 motor–cargo relationships, including motor activation as well as their number and organisation on cargoes. This is in part due to the lack of well-defined exemplary kinesin-1 cargoes, and the challenge of detecting kinesin-1 on moving cargoes using fluorescence-based imaging methods.

Kinesin-1 is also used by a number of different viruses to enhance their replication cycles, especially during their egress from infected cells (Diefenbach et al., 2002; Dodding and Way, 2011; DuRaine et al., 2018; Jouvenet et al., 2004; Pegg et al., 2021; Rietdorf et al., 2001; Strunze et al., 2011). Understanding how viruses recruit kinesin-1 via a limited set of proteins offers a great opportunity to understand the molecular basis of motor recruitment and regulation, as well as their organisation on a defined cargo. We previously demonstrated that during vaccinia virus infection, intracellular enveloped viruses (IEVs) recruit kinesin-1 to mediate their microtubule-dependent transport from their perinuclear site of assembly to the plasma membrane (Rietdorf et al., 2001). Disruption of the ability of IEVs to recruit kinesin-1 leads to a dramatic reduction in viral transport to the plasma membrane and cell-to-cell spread of the virus (Rietdorf et al., 2001; Ward and Moss, 2001). Kinesin-1 is recruited to IEVs by the interaction of A36, an integral IEV membrane protein with the tetratricopeptide repeats (TPRs) of the kinesin light chain (Ward and Moss, 2004). A36 interacts with the TPRs via a bipartite tryptophan acidic motif, which is present in many cellular proteins that bind kinesin-1 (Dodding et al., 2011; Pernigo et al., 2013). More recently, the viral E2/F12 complex, which associates with IEVs moving on microtubules (Dodding et al., 2009), was shown to enhance kinesin-1 binding to A36, suggesting that the virus also regulates motor recruitment (Carpentier et al., 2015; Gao et al., 2017).

However, in infected cells, IEVs only comprise a small proportion of total cytoplasmic virions compared to their precursor, the intracellular mature virus (IMV), the first infectious form of vaccinia virus assembled during infection (Carpentier et al., 2017; Leite and Way, 2015; Payne and Kristenson, 1979). Although abundant, IMVs are released late in the replication cycle when infected cells undergo lysis. This contrasts with IEVs, which fuse with the plasma membrane prior to cell lysis. IEVs are formed when IMVs acquire an additional membrane cisterna from the trans-Golgi network (TGN) or early endosomes (Leite and Way, 2015; Schmelz et al., 1994; Tooze et al., 1993). This envelopment results in the outer surface of IEVs having a very different composition of viral proteins from that of the IMVs, including the presence of A36 (Smith et al., 2002). Previous analysis demonstrates that IMVs can move at velocities up to 3 µm/s and are susceptible to nocodazole treatment, strongly implicating microtubules in their transport (Sanderson et al., 2000; Ward, 2005). It is thought that this motility is important to transport IMVs from their perinuclear site of assembly towards the TGN to facilitate membrane envelopment and IEV formation (Sanderson et al., 2000; Ward, 2005). In addition, microtubule transport of IMVs to the cell periphery might play a role in the cell-to-cell spread of vaccinia as IMVs are also capable of directly budding at the plasma membrane (Meiser et al., 2003; Tsutsui, 1983). Until now, the movement of IMVs on microtubules has not been imaged directly and the identity of the motor(s) responsible for their translocation to the TGN or plasma membrane remains to be established. Given this, we set out to identify the motor responsible for IMV transport using complementary *in vitro* and cell-based assays. Our analysis revealed that IMVs recruit kinesin-1, albeit at significantly lower levels than IEVs. Moreover, kinesin-1 is the major motor driving IMV motility *in vitro* and its loss leads to a significant defect in virion spread during infection.

## RESULTS

### IMVs undergo plus end-directed microtubule motility

**Fig. 1.**
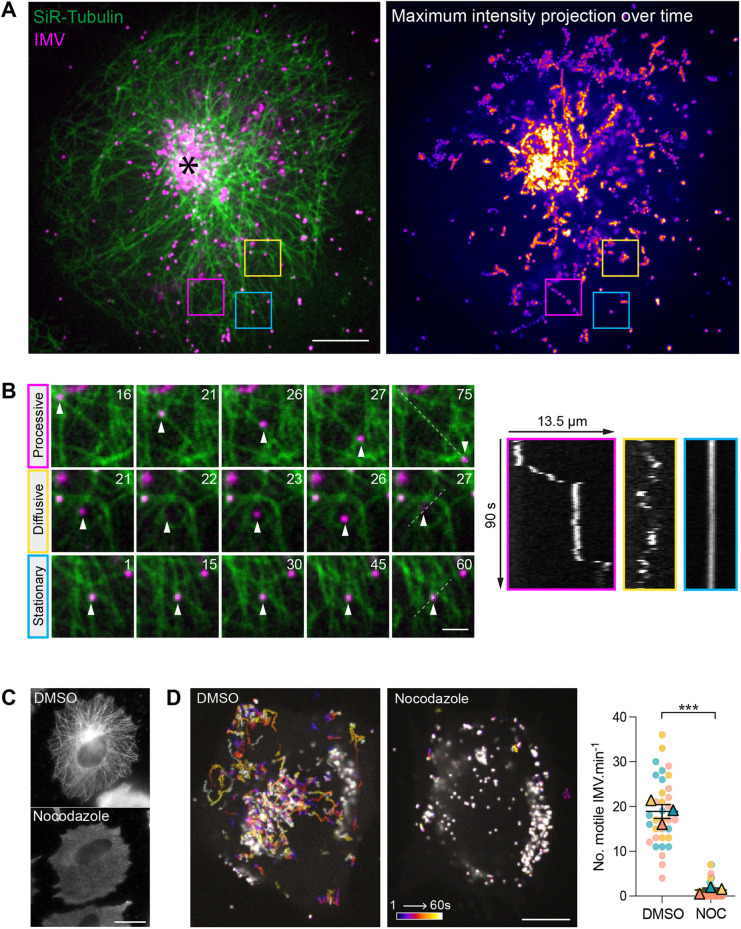
**IMVs undergo microtubule-based motility in cells.** (A) A representative image from a time-lapse movie showing a HeLa cell labelled with SiR-tubulin (green) at 7.5 h post infection with the ΔB5 RFP–A3 virus (magenta) to visualise microtubules and IMVs, respectively (see Movie 1). The asterisk indicates the perinuclear site of IMV assembly. Coloured boxed regions are enlarged in B. The maximum-intensity projection of the IMV channel over 90 s is shown on the right. Scale bar: 20 µm. (B) Enlarged boxed regions from A illustrate examples of processive, diffusive and stationary IMV (magenta) movements on microtubules (green) (see Movie 2). The time in seconds is indicated. The corresponding kymographs (shown on the right) for each IMV motion over 90 s were generated from the dotted lines as indicated. Scale bar: 2 µm (left). (C) Representative immunofluorescence images showing the organisation of microtubules using an anti-tubulin antibody in HeLa cells infected with the ΔB5 virus for 7.5 h and treated with DMSO or 33 µM nocodazole for 1 h. Scale bar: 10 µm. (D) Representative maximum-intensity projection images showing the movement of IMVs in HeLa cells infected with ΔB5 RFP–A3 for 7 h and treated with DMSO or 33 µM nocodazole for 1 h prior to imaging. IMV movement over 60 s is indicated by the timestamp bar (see Movie 3). Scale bar: 10 µm. (E) SuperPlot quantifying the numbers of motile IMVs (defined as IMVs travelling >3 µm) during the 60 s imaging window in infected cells treated with DMSO or 33 µM nocodazole for 1 h. *n*=34 cells per condition from three independent experiments. Data show the mean±s.e.m. Two-tailed unpaired Student's *t*-test was used to determine statistical significance. ***P≤0.001.

**Fig. 2.**
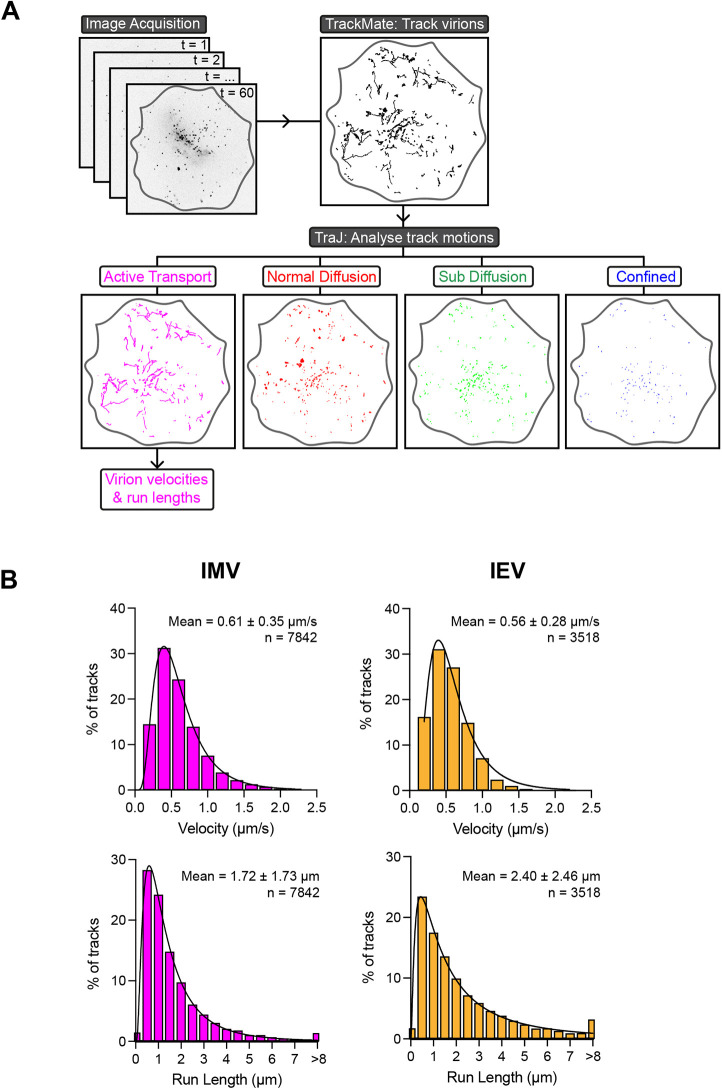
**Analysis of microtubule-based motility of IMVs in cells.** (A) Schematic of the image acquisition and analysis pipeline used to track virions and categorise their constituent movements as either active motion, normal diffusion, sub-diffusion or confined using Trackmate and TraJ. (B) Histograms of the velocities and run lengths of IMVs and IEVs undergoing active motion using automated tracking and analysis. *n*=7842 IMV and 2518 IEV runs from 15 ΔB5- and 22 WR-infected cells, respectively, from three independent experiments. Values show the mean±s.d.

To analyse IMV motility, we infected HeLa cells with the recombinant vaccinia strain ΔB5 which does not express B5, which is essential for IEV formation (Engelstad and Smith, 1993; Wolffe et al., 1993). The strain also encodes the RFP-tagged core protein A3 for visualisation (Arakawa et al., 2007). Live-imaging of ΔB5 RFP–A3 infected cells labelled with SiR-tubulin revealed that IMVs undergo a variety of movements; these include linear transport along microtubules (MTs), diffusion within the MT network and static association with MTs (Fig. 1A,B; Movies 1 and 2). Moreover, disrupting the MT network with nocodazole resulted in loss of IMV motility (Fig. 1C,D; Movie 3), in agreement with previous observations (Ward, 2005). To analyse IMV movements in detail, we performed automated single-particle tracking of fluorescently labelled virions using TrackMate (Fig. 2A) (Tinevez et al., 2017). Periods of active virion transport were discriminated from phases of diffusive and/or confined motion within each trajectory using TraJ (Fig. 2A) (Wagner et al., 2017). Using this approach, quantitative analysis of IMV sub-trajectories undergoing active transport revealed that they moved at an average velocity of 0.61±0.35 µm/s (mean±s.d.) over an average run length of 1.72±1.73 µm (Fig. 2B). Previous manual tracking of 65 virions suggested that IEVs move both faster and further, with an average velocity of 0.88±0.04 µm/s and run length of 6.44±0.37 µm (Dodding et al., 2011). However, automated tracking of a significantly larger number of IEVs using TrackMate revealed that they moved at 0.56±0.28 µm/s, which was similar to the velocity of IMVs, although IEVs still had longer average run lengths (2.40±2.46 µm) in cells (Fig. 2B).

**Fig. 3.**
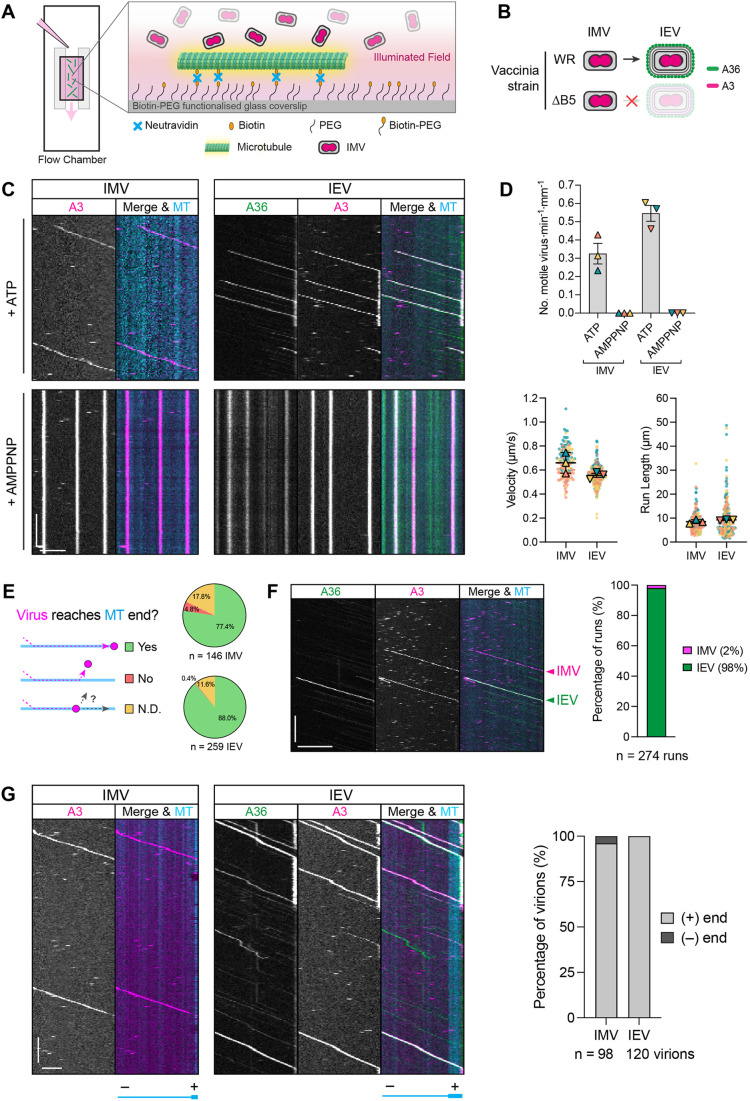
**Analysis of microtubule-based IMV and IEV movements *in vitro*.** (A) Schematic of an *in vitro* flow chamber illustrating the attachment of biotin-labelled and fluorescently labelled microtubules to a biotin–PEG-functionalised glass coverslip via a neutravidin link. RFP-tagged IMVs were visualised following addition of infected cell extracts into the chamber. (B) Schematic of the intracellular virions produced by wild-type Western Reserve (WR) or recombinant ΔB5 strains. Intracellular mature virions (IMVs) were labelled by RFP–A3 only, whereas intracellular enveloped virions (IEVs) were identified by RFP–A3 and A36–YFP markers. (C) Example kymographs of IMV or IEV movements on GMPCPP-stabilised microtubules (cyan) in the presence of 2 mM ATP or AMPPNP (see Movies 4 and 5). Scale bars: 30 s (vertical) and 5 µm (horizontal). (D) SuperPlots showing IMV and IEV *in vitro* motility rate in the presence of ATP or AMPPNP, and IMV and IEV velocities and run lengths in the presence of ATP. Error bars represent the mean±s.e.m. from three independent experiments in which 146 IMVs and 259­­ IEVs were analysed. (E) Pie charts showing the percentage of IMVs or IEVs that translocated to the end of the microtubule (MT). The percentage of virions that did not reach the end, or their fates were not discernible (N.D.) are also indicated. (F) Kymographs showing IMV and IEV movement along the same microtubule (MT) *in vitro* using extracts from HeLa cells infected with WR A36-YdF–YFP RFP–A3. Bar graph (right) shows the percentage of motile IMVs and IEVs. *n*=274 virus runs from three independent experiments. Scale bars: 30 s (vertical) and 10 μm (horizontal). (G) Kymographs of IMVs or IEVs moving on polarity-marked microtubules (cyan) *in vitro* (see Movies 6 and 7). Microtubule plus (+) and minus (−) ends are indicated below the images. The bar graph (right) shows the percentage of IMVs and IEVs moving towards microtubule (+) or (−) ends. *n*=98 IMVs or 120 IEVs from three independent experiments. Scale bars: 30 s (vertical) and 5 µm (horizontal).

The directionality of IMV movements is hard to assess in cells, as the dense microtubule network, especially near the nucleus, makes it difficult to determine whether virions are moving on single or bundled microtubules. The typical radial microtubule organisation is also disrupted during vaccinia infection, which also compounds the challenge of determining microtubule polarity and direction of transport (Ploubidou et al., 2000). Reconstitution of microtubule-based motility *in vitro* has provided major insights into the properties and regulation of kinesin-1 (Block et al., 1990; Chiba et al., 2022; Friedman and Vale, 1999; Hooikaas et al., 2019; Jiang et al., 2019; Seitz and Surrey, 2006; Svoboda et al., 1993). Moreover, microtubule-dependent transport of herpes simplex virus in cell extracts has been reconstituted *in vitro* (Lee et al., 2006; Wolfstein et al., 2006). Given this, and to overcome the issues of microtubule organisation in vaccinia-infected cells, we established an *in vitro* assay to analyse IMV motility on purified single microtubules using extracts from ΔB5 RFP–A3-infected HeLa cells (Fig. 3A). In parallel, we also analysed the movement of IEVs, which are distinguished from IMVs by the presence of A36, using extracts from cells infected with the Western Reserve (WR) strain of vaccinia expressing A36-YdF–YFP RFP–A3 (Fig. 3B). The A36-YdF recombinant virus was used as it is deficient in actin-based motility, whereas microtubule-based transport is unaffected (Rietdorf et al., 2001; Ward and Moss, 2001). We observed that both IMVs and IEVs could move along GMPCPP-stabilised microtubules in the presence of ATP but not in the presence of the non-hydrolysable ATP analogue AMPPNP (Fig. 3C,D; Movies 4 and 5). Consistent with our cell-based observations, IMVs and IEVs moved at an average velocity of 0.66±0.14 and 0.56±0.08 µm/s, respectively (Fig. 3D). Both viruses had similar run lengths averaging ∼8–9 µm and usually reached the microtubule end where they sometimes remained stationary rather than detaching (Fig. 3D,E; Movies 4 and 5). IMVs and IEVs were capable of moving on the same microtubule; however, the frequency of IEV movement was much higher than IMVs when both types of virions were present in the infected cell extract (Fig. 3F).

Interestingly, IMVs and IEVs always translocated towards one microtubule end and were never observed moving bidirectionally or travelling in opposite directions on the same microtubule, suggesting that they move exclusively to either the plus or minus ends. The *in vitro* unidirectional motility of IEV is likely towards the plus ends given that they recruit kinesin-1 in infected cells (Carpentier et al., 2015; Dodding et al., 2011; Rietdorf et al., 2001; Ward and Moss, 2004). *In vitro* assays using polarity-marked microtubules with bright plus ends confirmed that this was indeed the case (Fig. 3G; Movie 6). IMVs moved towards the microtubule plus ends in 96% of runs, suggesting that their transport is also driven by a kinesin (Fig. 3G; Movie 7). It is likely that the 4% of IMVs moving to the minus ends were false positives due to mislabelling of microtubule plus ends resulting from microtubule shearing and reannealing events during their preparation (Fallesen et al., 2017).

### Kinesin-1 is recruited to IMVs and IEVs

**Fig. 4.**
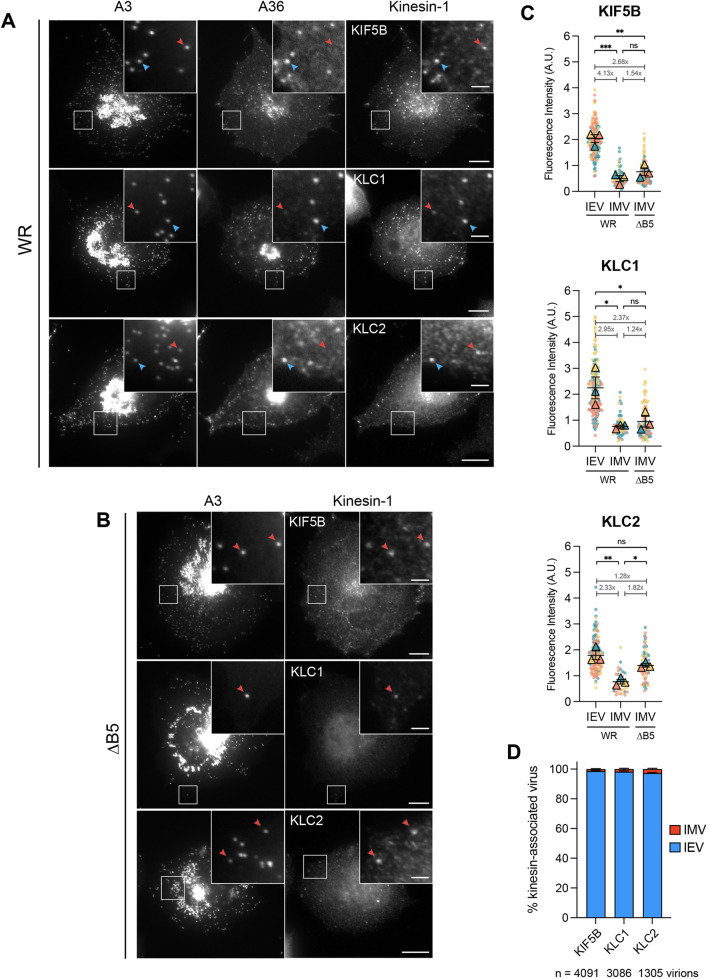
**IMVs and IEVs recruit endogenous kinesin-1 in infected cells.** (A,B) Representative immunofluorescence images of HeLa cells infected with (A) WR RFP–A3 or (B) ΔB5 RFP–A3 viruses and labelled with antibodies against A36 and/or KIF5B (top panels), KLC1 (middle panels) or KLC2 (bottom panels) 7.5 h post infection. Boxed regions highlight IEVs (blue arrowheads) or IMVs (red arrowheads) associated with kinesin-1. Scale bars: 10 µm and 2 µm (inset). (C) SuperPlots showing background-subtracted fluorescence intensities of antibodies against KIF5B, KLC1 or KLC2 associated with IEVs or IMVs in WR- or ΔB5-infected HeLa cells 7.5 h post infection. The fold differences between the means of each dataset are indicated. Error bars represent mean±s.e.m. from three independent experiments (*n*=33–174 measurements for each condition). Tukey's multiple comparison test was used to determine statistical significance. A.U., arbitrary units. ns, not significant, *P*>0.05; *P≤0.05; **P≤0.01; ***P≤0.001. (D) Bar graph showing the percentage of KIF5B-, KLC1- or KLC2-associated virions in WR-infected cells that were either IEVs or IMVs. Error bars represent mean±s.e.m. from three independent experiments. *n*=1305–4091 kinesin-associated virions from 32–35 cells.

Our observations with polarity-marked microtubules prompted us to assess whether kinesin-1 associates with IMV in infected cells. Immunofluorescence analysis of WR-infected HeLa cells revealed that IMVs and IEVs (identified by the absence or presence of A36, respectively) recruited endogenous KIF5B, KLC1 and KLC2 (Fig. 4A). Furthermore, endogenous kinesin-1 heavy and light chains associated with IMVs in ΔB5-infected cells (Fig. 4B). Quantification revealed that 4.6±0.7% of IMVs (A3 positive) recruited KIF5B in ΔB5-infected cells. Strikingly, the fluorescence intensity of endogenous kinesin-1 appeared brighter on IEVs compared to that on IMVs (Fig. 4A). Quantification of heavy-chain fluorescence intensity on virions in WR-infected cells demonstrated that IEVs recruited 4.13-fold more KIF5B than IMVs, despite the latter making up the majority of virions assembled during infection (Fig. 4C). The levels of KLC1 and KLC2 associated with IEVs were also significantly higher than those for IMVs, although the difference was not as great as that seen for KIF5B (Fig. 4C). Curiously, IMVs produced in ΔB5-infected cells recruited greater levels of KLC2 compared to IMVs in WR-infected cells (Fig. 4C). However, no significant difference was observed for the recruitment of KIF5B or KLC1. Quantification of motor recruitment revealed that 97–99% of all virions with kinesin-1 were IEVs (Fig. 4D), even though IMVs represent ∼80% of total intracellular viruses (Carpentier et al., 2017).

**Fig. 5.**
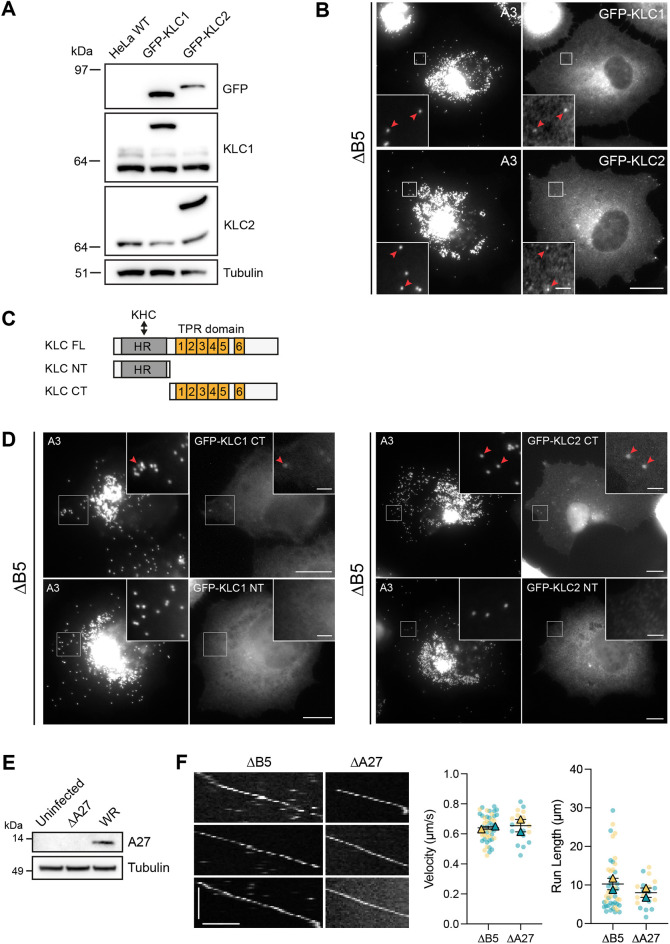
**IMVs recruit kinesin via the KLC TPR domains.** (A) Immunoblot analyses with the indicated antibodies of total cell lysates from parental HeLa wild-type (WT) or HeLa cells stably expressing of GFP-KLC1 or GFP-KLC2. (B) Representative immunofluorescence images showing HeLa cells stably expressing GFP-tagged KLC1 or KLC2 and infected with the ΔB5 RFP–A3 virus. Red arrowheads highlight IMV colocalisation with KLC. Scale bars: 10 µm and 2 µm (inset). (C) Schematic of KLC full length (FL), N-terminal (NT) and C-terminal (CT) constructs. The NT contains the heptad repeat (HR) domain, which binds kinesin heavy chain (KHC), whereas the CT contains the tetratricopeptide repeat (TPR) domain involved in cargo binding. (D) Representative immunofluorescence images showing HeLa cells transiently expressing the indicated GFP-tagged KLC construct and infected with ΔB5 RFP–A3 for 7.5 h. Insets show IMV colocalisation (red arrowheads) with the C-terminal (CT) domains of GFP-KLC1 and -KLC2 but not their N-terminal (NT) domain. Scale bars: 10 μm and 2 μm (inset). (E) Immunoblot analyses of whole-cell lysates from uninfected, ΔA27- or WR-infected HeLa cells using the indicated antibodies. (F) Example kymographs showing *in vitro* IMV motility in extracts derived from cells infected with ΔB5 RFP–A3 (left) or ΔA27 YFP–A4 (right) viruses. Scale bars: 20 s (vertical) and 5 μm (horizontal). The corresponding SuperPlots show IMV velocities and run lengths using these two virus strains. Bars represent mean±s.e.m. *n*=46 (ΔB5) or 19 (ΔA27) virions from two independent experiments. All images are representative of two independent experiments.

A36 recruits kinesin-1 to IEVs by interacting with the KLC TPRs (Dodding et al., 2011; Gao et al., 2017; Rietdorf et al., 2001; Ward and Moss, 2004). Given this, we examined whether IMVs also recruit kinesin-1 through a similar interaction. IMVs were capable of recruiting GFP-tagged KLC1 and KLC2 in ΔB5-infected cells (Fig. 5A,B). They could also recruit the C-terminal region of the light chain, which contains the TPRs, but not the N-terminal heavy chain-binding domain (Fig. 5C,D).

To date, A27 is the only IMV membrane protein that has been implicated in virion transport as its loss (using a virus with inducible A27 expression) resulted in the absence of IMV dispersion away from their perinuclear site of assembly (Sanderson et al., 2000). However, there is conflicting evidence, as IMVs are still motile in infected cells when the A27 gene is deleted (Ward, 2005). To investigate whether A27 is required for IMV transport, we performed *in vitro* motility assays using the ΔA27 virus, which, like the ΔB5 strain, only produces IMVs (Ward, 2005). We found that loss of A27 had no impact on virion motility, indicating that A27 is not required for IMV transport *in vitro* (Fig. 5E,F).

### Kinesin-1 drives microtubule-dependent movement of IMVs and IEVs

**Fig. 6.**
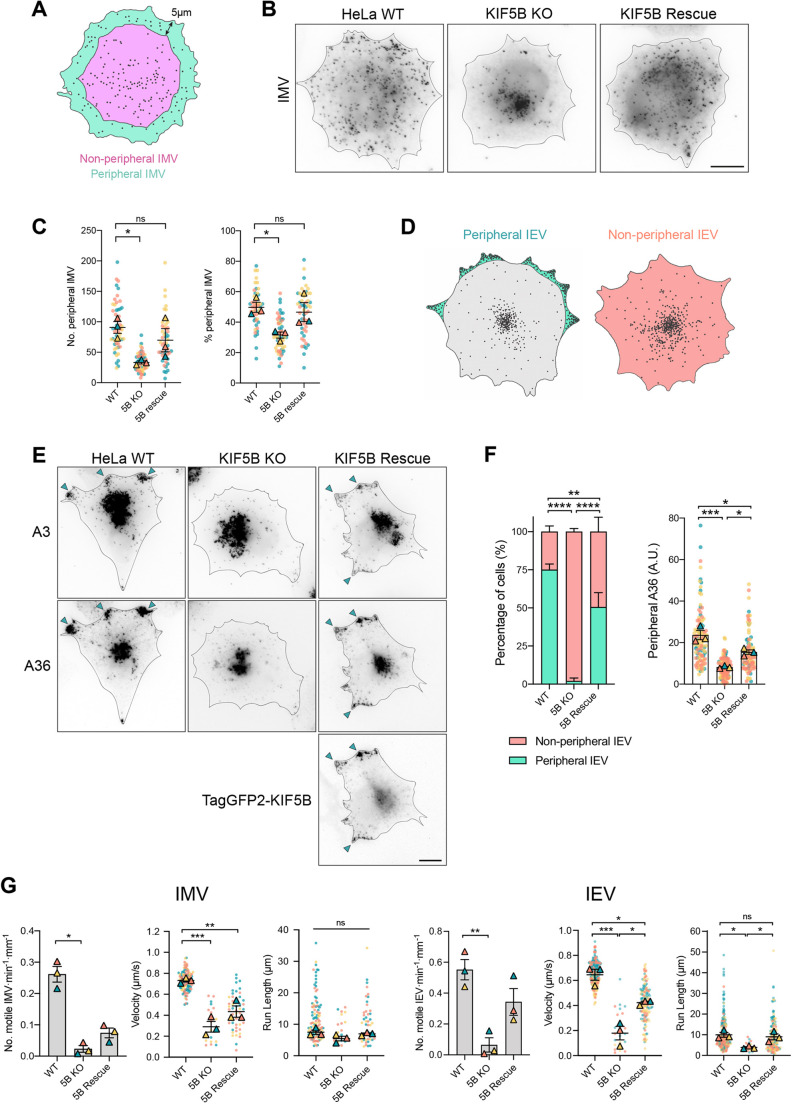
**Loss of kinesin-1 impairs IMV and IEV spread and motility.** (A) Schematic illustrating the area corresponding to the peripheral region <5 μm from the cell edge (teal) and non-peripheral area (pink) >5 µm from the cell edge. IMVs within each region were counted to determine the total number and proportion of IMVs reaching the cell periphery 7.5 h post infection. (B) Representative inverted immunofluorescence images showing dispersion of IMVs, labelled with an antibody detecting the IMV membrane protein A27, in the indicated cell lines at 7.5 h post infection with ΔB5 RFP–A3. Scale bar: 10 µm. (C) SuperPlots showing quantification of the number of peripheral IMVs (left) and the percentage of total IMVs (right) at the cell periphery in the indicated cell lines (KIF5B is indicated as 5B) from >50 cells in three independent experiments. Error bars represent the mean±s.e.m. Dunnett’s multiple comparisons test was used to determine statistical significance. (D) Illustration showing the accumulation of IEVs at the perinuclear region and cell vertices (shaded green, left cell) or lack of accumulation at the cell vertices (shaded pink, right cell). (E) Representative inverted immunofluorescence images labelled with the indicated markers showing IEV spread in the indicated cell lines 7.5 h post infection with WR A36-YdF RFP–A3 virus and labelled with anti-A36 antibody. The arrowheads indicate the accumulation of IEVs at cell peripheries. Scale bar: 10 µm. (F) Bar graphs showing the percentages of cells with peripheral IEV accumulation (left) and quantification of IEV spread to the cell periphery (right) based on fluorescence intensity of the anti-A36 antibody. Error bars represent mean±s.e.m. from >50 cells in three independent experiments. Tukey's multiple comparisons test was used to determine statistical significance. A.U., arbitrary units. (G) SuperPlots of the *in vitro* motility rates, velocities and run lengths for IMVs (*n*=116, 18 and 48) and IEVs (*n*=227, 23 and 124) in extracts of the indicated infected cells. Error bars represent mean±s.e.m. from three independent experiments. Tukey's multiple comparisons test was used to determine statistical significance. ns, not significant, *P*>0.05; *P≤0.05; **P≤0.01; ***P≤0.001; ****P≤0.0001.

To explore the involvement of kinesin-1 in IMV motility, we infected a kinesin-1 knockout (KO) HeLa cell line generated by CRISPR/Cas9 targeting of the *KIF5B* gene (Jia et al., 2017) as well as a KIF5B-rescued line stably expressing TagGFP2–KIF5B (Fig. S1A). To assess the role of kinesin-1 in IMV transport, we quantified the total number and proportion of IMVs that reached within 5 µm of the cell periphery in cells with or without KIF5B (Fig. 6A). This direct comparison was possible as there was no significant difference in cell size in the presence or absence of KIF5B (Fig. S1B). Our analysis revealed that IMV spread was impaired in the absence of KIF5B as significantly fewer IMVs reached the cell periphery (Fig. 6B,C), despite the ability of IMVs to slowly disperse by random diffusion (Sodeik, 2000). Importantly, this defect was rescued by the stable expression of TagGFP2–KIF5B in the KIF5B KO cell line (Fig. 6B,C; Fig. S1A). In parallel, we analysed the impact of the loss of kinesin-1 on IEV transport to the cell periphery using the A36-YdF RFP–A3 virus, which is deficient in actin-based transport (Fig. 6D) (Rietdorf et al., 2001; Ward and Moss, 2001). We found that in the absence of kinesin-1, there was a dramatic reduction in the percentage of cells with peripheral IEV accumulations, a phenotype rescued by the expression of TagGFP2–KIF5B (Fig. 6E,F). Furthermore, TagGFP2–KIF5B colocalised with IEVs at the cell periphery in the KIF5B-rescued cell line (Fig. 6E).

To extend these observations, we analysed IMV and IEV motility *in vitro* using extracts from infected parental, KIF5B KO or KIF5B-rescued HeLa cells. Strikingly, there was a 91% reduction in microtubule-based transport of IMVs in extracts lacking KIF5B compared to the parental HeLa control (Fig. 6G). In the absence of kinesin-1, the few motile IMVs had a 63% reduction in velocity – 0.28±0.05 µm/s compared to 0.73±0.01 µm/s for the control (Fig. 6G). The partial recovery of this phenotype in the KIF5B-rescued cells might be due to the reduced concentration of kinesin-1 in the extract because of the low expression of TagGFP2–KIF5B in the rescued cells (Fig. S1A). Similarly, IEVs displayed negligible rates of motility and reduced velocities and run lengths in the absence of KIF5B, which were also partially rescued by the presence of TagGFP2–KIF5B in extracts from A36-YdF–YFP RFP–A3 infected cells (Fig. 6G). Taken together, our observations demonstrate that kinesin-1 mediates the spread of both IMVs and IEVs from their perinuclear sites of assembly to the cell periphery.

### IEVs and IMVs recruit large but differing numbers of kinesin-1 motors

**Fig. 7.**
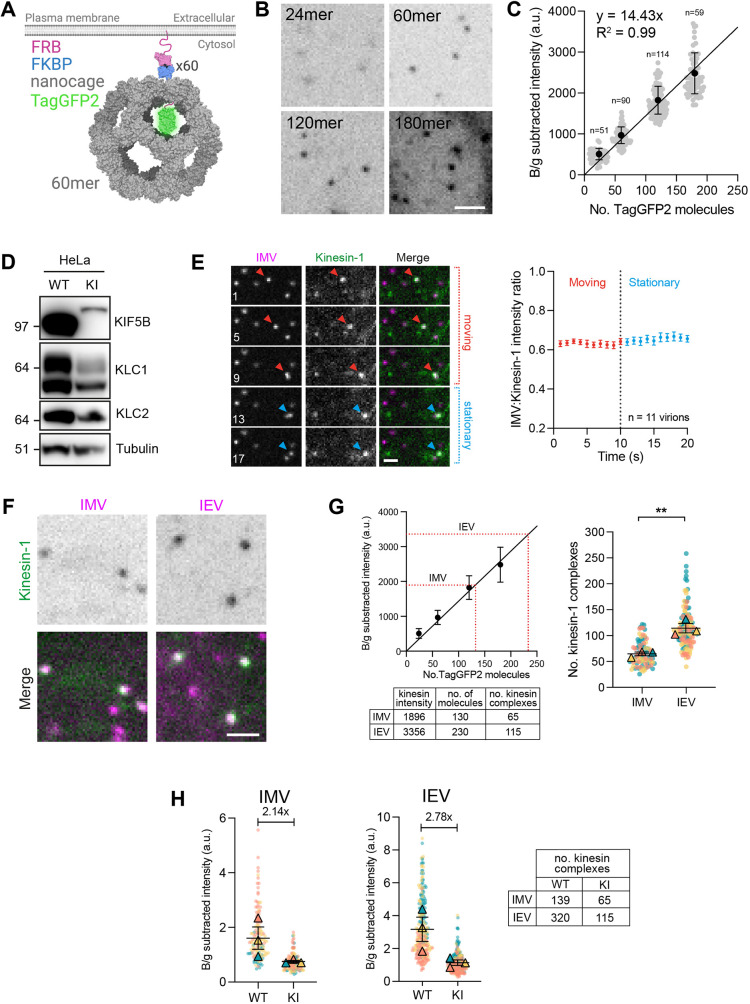
**Quantifying the number of kinesin-1 complexes on IMVs and IEVs.** (A) Schematic of the intracellular TagGFP2-tagged 60-mer nanocage. Each subunit of the nanocage (grey) is fused with TagGFP2 (green) and FKBP (blue), although this is shown only for one subunit for clarity. FRB (pink) is targeted to the plasma membrane by its palmitoylation and myristoylation sequence and dimerises with FKBP in the presence of the rapamycin analogue AP21967. PDB structures used: 5KP9, 2Y0G and 4DRI. (B) Representative average-intensity projection images of transiently expressed TagGFP2-tagged nanocages in HeLa cells treated with 500 nM AP21967. Scale bar: 2 µm. (C) Quantification of fluorescence intensities of TagGFP2-tagged 24-, 60-, 120- and 180-mer nanocages. Error bars represent mean±s.d. Linear line of regression is fitted. *n*=51–114 measurements per nanocage from three independent experiments. (D) Immunoblot analysis of total cell lysates from HeLa wild-type (WT) or TagGFP2–KIF5B CRISPR knock-in (KI) cells using the indicated antibodies. (E) Representative images from time-lapse movie showing the association of kinesin-1 (green) with IMVs (magenta) during moving (red arrowheads) and stationary (blue arrowheads) phases in the HeLa TagGFP2–KIF5B knock-in cell line at 7.5 h post infection with the ΔB5 RFP–A3 virus (see Movie 8). Time in seconds is indicated in each image. Scale bar: 2 µm. The graph on the right shows quantification of the TagGFP2–KIF5B:RFP–A3 fluorescence intensity ratio on IMV particles during moving and stationary phases. *n*=11 virions from two independent experiments. (F) Representative average-intensity projections of endogenously expressed TagGFP2–KIF5B (green) on IEVs or IMVs (magenta) in HeLa TagGFP2–KIF5B knock-in cells 7.5 h post infection with ΔB5 RFP–A3 (left) or WR B5-RFP (right). Scale bar: 2 µm. (G) The left graph shows the mean background-subtracted fluorescence intensity of TagGFP2–KIF5B together with the calculated number of molecules on IMVs and IEVs, superimposed (dotted red lines) on the nanocage calibration plot from C. The table below shows the summary of the readout. SuperPlot (right) showing the number of kinesin-1 complexes associated with IMVs or IEVs from three independent experiments in which 84 and 121 virions were analysed for IMVs and IEVs, respectively. Bars represent mean±s.e.m. Two-tailed unpaired ­Student's *t*-test was used to determine statistical significance. **P≤0.01. (H) SuperPlots showing the background-subtracted antibody intensity signals of KIF5B associated with IMVs (left graph) or IEVs (right graph) in HeLa wild-type (WT) or tagGFP2–KIF5B knock-in (KI) cells. The fold difference between the mean number of KIF5B associated with virions in WT or KI cells is shown. The table summarises the mean number of kinesin-1 complexes associated with IMVs or IEVs in HeLa WT or KI cells after correcting for low levels of tagGFP2–KIF5B expression in the latter. a.u., arbitrary units.

Our immunofluorescence analysis suggests that IMVs and IEVs recruit different numbers of kinesin-1 motors (Fig. 4C). Given that the absolute number of kinesin-1 motors on a bona fide cellular cargo remains to be established, we set out to determine the number of kinesin-1 complexes recruited to IMVs and IEVs. To achieve this, we used a similar approach as Akamatsu et al. (2020) by taking advantage of self-assembling protein nanocages (Hsia et al., 2016). When expressed in cells, the assembled nanocages have a defined number of TagGFP2 molecules (Fig. 7A). Addition of the rapamycin analogue AP21967 promotes a FKBP–FRB-dependent tethering of the nanocages to the plasma membrane, limiting their diffusion. Imaging the nanocages and quantifying their fluorescence intensities allowed us to generate a calibration curve that could be used to determine the number of TagGFP2-tagged kinesin-1 motors recruited to virions. To extend the previous calibration curve, we generated an additional nanocage with 180 TagGFP2 molecules, then measured its background-subtracted fluorescence intensity together with the previously described 24-, 60- and 120-mer nanocages (Akamatsu et al., 2020) using spinning-disc confocal microscopy (Fig. 7B). The average fluorescence intensity values were proportional to the predicted numbers of TagGFP2 per nanocage, including those of the new 180-mer species (Fig. 7C).

To compare the fluorescence intensities of TagGFP2-tagged nanocages with kinesin-1 associated with IMVs or IEVs, we generated an endogenously expressed TagGFP2–KIF5B HeLa knock-in cell line by CRISPR/Cas9 genome editing and single-cell cloning (Fig. S1C). As with our previous TagGFP2–KIF5B rescue cell line, immunoblot analysis showed that TagGFP2–KIF5B expression was reduced in the knock-in cell line compared to that of the untagged motor in the parental cells (Fig. 7D). Nevertheless, all kinesin-1 motors in the knock-in cell line were fluorescently tagged and amenable for analysis. As the molecule counting method requires imaging *z*-stacks, the fast microtubule-based movements of IMVs and IEVs presented a challenge for capturing the intensity of TagGFP2–KIF5B on moving virions due to the temporal constraints. Live-cell imaging, however, revealed that the fluorescence intensity ratio between viral RFP–A3 and TagGFP2–KIF5B signals in a single *z*-plane did not significantly change between phases of IMV motility and pausing (Fig. 7E; Movie 8). We therefore quantified the number of kinesin-1 molecules on stationary IMV and IEV particles in TagGFP2–KIF5B HeLa knock-in cells infected with ΔB5 RFP–A3 or WR B5–RFP, respectively. In agreement with our previous immunofluorescence analysis, we found that IEVs recruit more kinesin-1 than IMVs (Fig. 7F,G). Comparison of the fluorescence intensities of TagGFP2–KIF5B on each virion with our nanocage calibration curve revealed that IMVs and IEVs recruited an average of 130±8 and 230±18 (mean±s.e.m.) KIF5B molecules, which is equivalent to 65±4 and 115±9 kinesin-1 motor complexes, respectively (Fig. 7G). We wondered, however, whether these values are an underestimate given the significantly lower expression level of TagGFP2–KIF5B compared to that of the untagged motor in the parental cells (Fig. 7D). To examine whether this was the case, we performed immunolabelling with the KIF5B antibody and quantified the fluorescence intensity of the KIF5B signal associated with IMVs and IEVs in the parental HeLa and TagGFP2–KIF5B HeLa knock-in cells. In both cases, the KIF5B signal was stronger in the parental cells compared to that in the knock-in cell line, being 2.14 and 2.78-fold higher on IMVs and IEVs, respectively, which corresponds to an average of 139 and 320 motor complexes for the two different viruses (Fig. 7H).

**Fig. 8.**
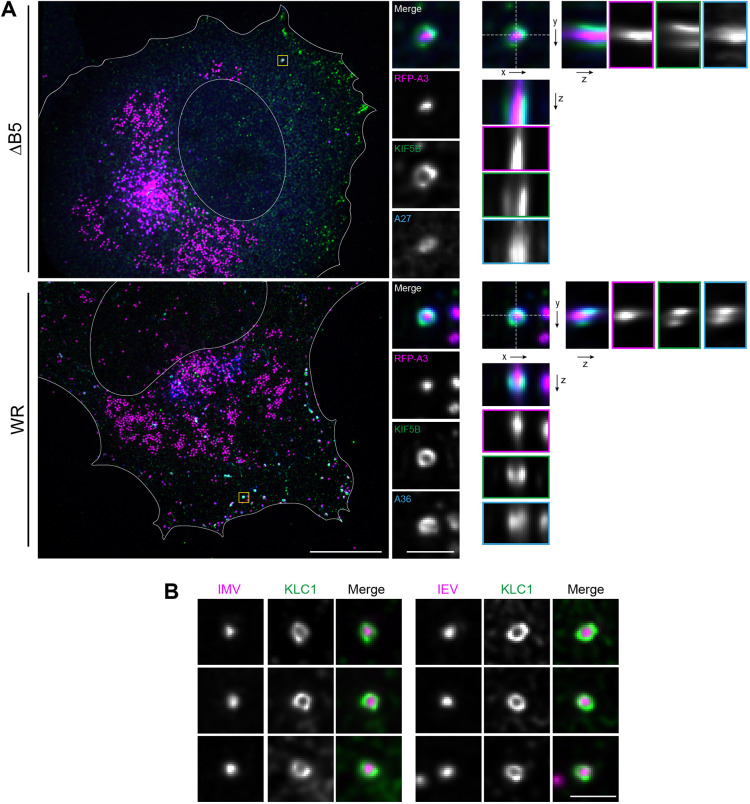
**Super-resolution imaging of kinesin-1 associated with virus particles.** (A) Maximum-intensity projections of deconvolved super-resolution images of a HeLa cell infected with ΔB5 RFP–A3 (upper panel) or WR RFP–A3 (lower panel) and immunolabelled for KIF5B (green) and either A27 (blue) or A36 (blue) as indicated. Boxed regions are enlarged on the right, along with the corresponding *xz* and *yz* orthogonal views. Dotted lines show the cross-section used. Scale bars: 10 µm and 1 µm (insets). (B) Maximum-intensity projections showing additional examples in which kinesin-1 (green), detected with the anti-KLC1 antibody, is present on IMVs and IEVs (magenta) at 7.5 h post infection with ΔB5 RFP–A3 or WR RFP–A3. Scale bar: 1 µm. Images are representative of two experiments.

It has been suggested that kinesin motors are likely to be clustered on cellular cargoes to ensure more efficient processive transport (Erickson et al., 2011). Given this, we wondered how the relatively large numbers of kinesin-1 motors were spatially organised on the surface of IMVs and IEVs. Super-resolution imaging of fixed ΔB5- and WR-infected cells using structured illumination microscopy combined with deconvolution revealed that kinesin-1 was distributed over the whole IMV or IEV surface (Fig. 8A,B).

## DISCUSSION

Many viruses are critically dependent on microtubule-driven transport during the establishment of infection as well as the egress of new viral progeny from their infected host in the absence of cell lysis (Dodding and Way, 2011; Greber and Way, 2006; Niehl et al., 2013; Radtke et al., 2006; Seo and Gammon, 2022; Walsh and Naghavi, 2019). In the case of vaccinia virus, which replicates in cytoplasmic perinuclear viral factories (Leite and Way, 2015), it has been calculated that it would take 5–6 h for newly assembled virions to diffuse 10 µm (Sodeik, 2000). Given this rate, combined with the random nature of diffusion, it is not surprising that vaccinia uses microtubule-dependent transport to reach the plasma membrane to enhance the efficiency of its spread. During vaccinia infection, intracellular enveloped viruses (IEVs) use kinesin-1-mediated microtubule transport to reach the plasma membrane from their perinuclear site of assembly (Rietdorf et al., 2001; Ward and Moss, 2001; Schepis et al., 2007). However, IEV formation depends on the prior assembly of intracellular mature viruses (IMVs) (Leite and Way, 2015; Smith et al., 2002), which can also undergo microtubule-dependent movement (Sanderson et al., 2000; Ward, 2005). We have now uncovered that IMVs also recruit kinesin-1 to move to the plus ends of microtubules.

### IEVs are more effective in recruiting kinesin-1 than IMVs

Previous observations missed that IMVs also recruit kinesin-1 (Carpentier et al., 2015; Gao et al., 2017; Rietdorf et al., 2001; Ward and Moss, 2004). The main reason for this omission is most likely owing to the fact that 97–99% of all intracellular viruses with kinesin-1 are IEVs (Fig. 4D). This observation is even more striking given that IMVs represent ∼80% of the total intracellular viruses (Carpentier et al., 2017). That IMVs are less effective in recruiting kinesin-1 is also consistent with our *in vitro* observations, in which IEVs accounted for ∼98% of all the virus runs in extracts from A36-YdF–YFP RFP–A3 infected cells (Fig. 3F). Taken together, our observations suggest that a competition exists between IEVs and IMVs for binding kinesin-1 during infection. In support of this notion, in the absence of IEVs in ΔB5 RFP–A3-infected cells, the levels of KIF5B, KLC1 and KLC2 all increased on IMVs (Fig. 3C). Our observations on the levels of kinesin-1 associated with the two different virions suggest that IEVs have more binding sites and/or greater affinity for the motor than IMVs.

To determine the absolute number of motors on IMVs and IEVs, we performed quantitative imaging using nanocages tagged with defined numbers of GFP molecules as internal standards following the approach of Akamatsu et al. (2020). Using this method, after correcting for the low levels of TagGFP2–KIF5B expression in our HeLa knock-in cells, we found that IMVs and IEVs recruit an average of 139 and 320 kinesin-1 motor complexes, respectively (Fig. 7). Based on the virion dimensions in frozen hydrated vaccinia-infected cells using cryo-electron tomography, we calculated that IMVs have a surface area of 238,446 nm^2^, whereas for IEVs, it is 405,037 nm^2^ (Hernandez-Gonzalez et al., 2022 preprint). This means that not only the total number of kinesin-1 motors is greater on IEVs than IMVs, but their density is also greater (one motor complex per 1265 nm^2^ for IEVs compared to 1715 nm^2^ for IMVs). This significant difference in motor number and density might explain why IEVs are more efficient (longer run lengths) than IMVs in their transport to the plasma membrane (Fig. 2B). Indeed, in previous experimental and theoretical studies, increasing the number of kinesin motors attached to a cargo leads to greater distances travelled along the microtubule (Beeg et al., 2008; Derr et al., 2012; Furuta et al., 2013; Korn et al., 2009; Muller et al., 2010; Vershinin et al., 2007). We have also previously observed that impairing the ability of IEVs to recruit kinesin-1 by mutating the A36 WD KLC-binding motif reduces the run length of IEVs from 6.44±0.37 to 2.58±0.14 µm without affecting viral speed (Dodding et al., 2011). Using our motor quantification approaches, we found that IEVs recruit an average of 72 rather than 320 kinesin-1 motor complexes when the A36 WD KLC-binding motif was mutated (Fig. S2).

Our motor number values are significantly larger than previous studies that typically observed 1–11 kinesin motors on a cargo using immunogold labelling in electron microscopy sections or were based on inferences from cargo velocities or force measurements (Ashkin et al., 1990; Gross et al., 2007; Hill et al., 2004; Kural et al., 2005). In contrast, our results showed that IMVs and IEVs recruited 139 and 320 kinesin-1 motors, respectively. Given the respective diameters of IMVs (350×280×200 nm) and IEVs (440×380×260 nm) (Hernandez-Gonzalez et al., 2022 preprint), our motor numbers are in line with previous simulations that suggest that 100 nm and 500 nm vesicles require 35 and 800 kinesin-1 motors, respectively, to travel distances >10 µm (Jiang et al., 2019).

It has been suggested that kinesin motors are likely to be clustered on cellular cargoes to ensure more efficient processive transport (Erickson et al., 2011). Structured illumination microscopy revealed, however, that kinesin-1 is distributed over the entire surface of IMVs and IEVs (Fig. 8). Such an organisation might help the virions navigate the dense cellular microtubule network by allowing them to quickly switch microtubule tracks and/or bypass roadblocks for efficient transport in the crowded cytosol (Lakadamyali, 2014; Tjioe et al., 2019). Furthermore, given this organisation, it is likely that only a subset of bound motors is active at any given time owing to the geometric constraints of motor positioning on the virion relative to a microtubule. Indeed, it is predicted that for a 100 nm vesicle with 35 bound kinesin-1 motors, three motors are sufficient to engage the microtubule and drive vesicle transport over distances of 10 µm (Jiang et al., 2019). However, determining the number of active motors on a cargo in live cells still remains a considerable challenge (Cai et al., 2007) as, although cargo binding relieves auto-inhibition, it might not always result in full motor activation (Blasius et al., 2007; Chiba et al., 2022; Fu and Holzbaur, 2013; Kawano et al., 2012; Twelvetrees et al., 2019) without additional regulation through post-translational modifications and/or microtubule-associated proteins (Hooikaas et al., 2019; Manser et al., 2012). Given our observations, we suggest that vaccinia-infected cells offer a powerful model system with which to develop and test sensors for the activation state of kinesin-1 on moving cellular cargoes.

### How do IMVs recruit kinesin-1?

Kinesin-1 is recruited to IEVs via an interaction between the TPRs of the kinesin light chain with A36, an integral membrane protein exposed on the surface of the virion (Rietdorf et al., 2001; Rottger et al., 1999; van Eijl et al., 2000; Ward and Moss, 2004). The TPR domain binds directly to a bipartite tryptophan acidic motif in A36, which is also found in many other cellular proteins that can bind kinesin-1 (Dodding et al., 2011; Pernigo et al., 2013). In addition, the viral E2/F12 complex, which is recruited to IEVs moving on microtubules (Dodding et al., 2009), also enhances kinesin-1 binding to A36, suggesting that the virus also regulates motor recruitment (Carpentier et al., 2015; Gao et al., 2017).

Notably, the IMV surface has a very different composition of viral proteins compared to that of the IEV, including the absence of A36 (Smith et al., 2002). Nevertheless, IMVs still recruit kinesin-1 via the KLC TPR (Fig. 5D). However, examination of the sequences of known IMV surface proteins failed to identify obvious W-acidic or Y-acidic motifs that mediate interactions with TPR repeats (Dodding et al., 2011; Pernigo et al., 2018, 2013; Yip et al., 2016). A lack of these motifs might point to a different interaction with the KLC TPR domain, such as that seen for JIP3 (Cockburn et al., 2018). What is clear from our analysis and in agreement with the observations of Ward (2005) is that A27, an abundant membrane protein on the surface of IMVs that has been implicated in their transport (Sanderson et al., 2000), is not required for microtubule-dependent transport of IMVs (Fig. 5E,F).

A36, which is not required for IEV formation (Rottger et al., 1999; Sanderson et al., 1998; Wolffe et al., 1998), was originally shown to be required for their microtubule-dependent transport using a deletion strain lacking the protein (Rietdorf et al., 2001; Ward and Moss, 2001). However, future work to identify the IMV protein responsible for kinesin-1 recruitment will not be so straightforward using a genetic approach. This is because the majority of IMV surface proteins are required for entry and/or IMV formation (for examples, see Lin et al., 2000; Unger et al., 2013; Wolfe et al., 2012).

### Other microtubule motors can drive virion motility

Kinesin-1 is clearly the major motor driving IMV and IEV motility. However, in the absence of kinesin-1, limited numbers of IMVs and IEVs are still weakly processive *in vitro*, suggesting that they might utilise additional kinesin member(s) for microtubule-based motility (Fig. 4G). The recruitment of multiple motor classes might help virions navigate the heterogenous microtubule network of the cell, as different kinesins have preferences for specific microtubule subsets marked by their post-translational modifications and/or microtubule-associated proteins. This has been well documented in neuronal cells (Hammond et al., 2010; Lipka et al., 2016) but has also been observed in non-neuronal cell types (Cai et al., 2009; Guardia et al., 2016). In line with this, kinesin-1 (KIF5B) and kinesin-3 (KIF13B) motors drive efficient transport of Rab6-positive vesicles along different microtubule populations to reach the cell periphery where they undergo exocytosis (Serra-Marques et al., 2020). In future studies, it will be interesting to resolve whether other kinesin members are also recruited by vaccinia virus and whether they cooperate with kinesin-1 to promote virion transport.

In conclusion, our study shows that kinesin-1 drives the transport and spread of both intracellular forms of vaccinia virus. In addition, we show for the first time that microtubule-based motility of both IMVs and IEVs can be reconstituted in infected cell extracts *in vitro*. This will no doubt provide a useful model system to obtain further insights into motor–cargo relationships and motor regulation. The task ahead is to uncover the mechanistic basis for kinesin-1 recruitment to IMVs and determine how motor recruitment and activation is regulated by IMVs and IEVs.

## MATERIALS AND METHODS

### Cells and generation of stable cell lines

HeLa cells, authenticated by STR profiling and mycoplasma-tested by the Francis Crick Institute Cell Service, were maintained in minimum essential medium (MEM, Sigma-Aldrich, M4655), or Dulbecco's modified Eagle medium (DMEM, Thermo Fisher Scientific, 41966) supplemented with 10% fetal bovine serum (Sigma-Aldrich, F7524), 100 U/ml penicillin and 100 µg/ml streptomycin at 37°C and 5% CO_2_. Stable HeLa cell lines expressing GFP–KLC1 and GFP–KLC2 were generated using lentivirus infection (Trono group second generation packaging system, Addgene) and selected using puromycin resistance (1 µg/ml) as previously described (Abella et al., 2016). The HeLa KIF5B KO cell line was kindly provided by Juan Bonifacino (National Institutes of Health, USA) (Jia et al., 2017). Lentiviral expression vectors were used to stably express TagGFP2–KIF5B in HeLa KIF5B KO cells to generate the HeLa KIF5B rescue cell line.

The vectors pLVX GFP-KLC1 and pLVX GFP-KLC2 were generated by sub-cloning the murine KLC1A and KLC2 coding sequences (Dodding et al., 2011) into the EcoRI/BamHI and NotI/EcoRI sites, respectively, of a pLVX N-terminal GFP parental vector (Abella et al., 2016). To generate the pLVX TagGFP2-KIF5B vector, the murine KIF5B coding sequence was amplified from a plasmid provided by Marvin Bentley (Rensselaer Polytechnic Institute, NY) (Yang et al., 2019), TagGFP2 was amplified from a plasmid provided by David Drubin (University of California, Berkeley, CA) (Akamatsu et al., 2020) and both inserted between the XhoI and EcoRI sites of the parental pLVX N-terminal GFP vector using Gibson assembly (New England Biolabs) following the manufacturer's instructions. These lentiviral vectors were used to establish stable HeLa cell lines as previously described (Weisswange et al., 2009). SnapGene software (Insightful Science; https://www.snapgene.com/) was used to plan and visualise cloning strategies and to analyse sequencing results.

### Expression constructs

The expression vector pEL KLC2-TPR has been described previously (Rietdorf et al., 2001). KLC sequences comprising residues 1–155 of KLC2 and residues 1–162 or 163–538 of murine KLC1A were amplified by PCR and cloned into the NotI/EcoRI site of the pEL N-terminal GFP parental vector using Gibson assembly (New England Biolabs) following the manufacturer's instructions (Rietdorf et al., 2001). The KLC1A and KLC2 coding sequences used for PCR amplification have been previously described (Dodding et al., 2011). The fidelity of all expression constructs was confirmed by sequencing.

### CRISPR/Cas9-mediated gene editing

The HeLa CRISPR-Cas9 knock-in cell line expressing TagGFP2–KIF5B at the endogenous KIF5B locus was generated using the pORANGE vector containing SpCas9 (Addgene plasmid #131471) (Willems et al., 2020). The guide RNA (gRNA) for KIF5B was designed using a CRISPR design webpage tool (https://www.benchling.com/). The targeting sequence used was 5′-CCCGGCTGCGAGAAAGATGG-3′ (coding strand sequence indicated). CRISPR/Cas9-mediated knock-in of TagGFP2 into the endogenous KIF5B locus was performed according to the protocol described in Willems et al. (2020). In brief, HeLa cells were transfected using JetPrime (Polyplus) with the pORANGE vector bearing the appropriate gRNA targeting sequence and TagGFP2 insert. The gRNA targets the ATG start codon of KIF5B exon 1 where Cas9 induces a double strand break. The TagGFP2 coding sequence is integrated into the incision site through repair by non-homologous end joining. After initial transfection, cells were allowed to recover for ∼3 weeks before single-cell colonies were isolated by fluorescence-activated cell sorting. Individual clones were screened for biallelic integration of TagGFP2 into the KIF5B loci by junction PCR and immunoblot analyses. Sequencing confirmed successful in-frame integration of the TagGFP2 sequence. The primers used for sequencing and PCR were 5′-CTCTCACGGCCCTCGCGACCACAAGCCCTCAG-3′ and 5′-AAACTTGGCGATGTACTTGTCGCCGCGGTTCACTT-3′.

### Recombinant viruses and infection

All recombinant viruses are generated in the Western Reserve (WR) strain of Vaccinia virus. The recombinant vaccinia virus strains RFP–A3 and ΔA27 YFP–A4 (kindly provided by Brian Ward, University of Rochester Medical Center, NY) have been previously described (Ward, 2005; Weisswange et al., 2009). The LA-RFP-A3-RA targeting vector was used to insert RFP–A3 as previously described (Weisswange et al., 2009) into the genome of the existing viral strains ΔB5 (Engelstad and Smith, 1993), A36-YdF (Rietdorf et al., 2001), and A36-YdF-YFP (Arakawa et al., 2007). The recombinant A36-YdF–YFP virus strains containing the WE/AA or WD/AA mutations were generated as previously described (Dodding et al., 2011). The recombinant strain expressing B5–RFP was generated by rescuing the ΔB5 virus with a B5–RFP targeting vector and isolating virus plaques with a plaque size that is similar to WR and expression of RFP.

For live- and fixed-cell imaging, HeLa cells on fibronectin-coated glass coverslips or glass-bottomed dishes were infected with the relevant recombinant vaccinia virus in serum-free MEM or DMEM at a multiplicity of infection of 1. After 1 h at 37°C, the serum-free media was removed and replaced with complete media. Cells were incubated at 37°C and, at 7.5 h post infection, cells were imaged live or were fixed and processed for immunofluorescence analysis. For nocodazole experiments, DMSO control or 33 µM nocodazole (Sigma-Aldrich, M1404) was added to culture medium for 1 h prior to fixation or live-cell imaging.

### Immunofluorescence and immunoblot analysis

HeLa cells were either fixed with 4% paraformaldehyde in PBS or ice-cold methanol for 10 min, permeabilised (for paraformaldehyde fixation) with 0.1% Triton X-100 in PBS for 5 min, then blocked in cytoskeletal buffer (10 mM MES, 150 mM NaCl, 5 mM EGTA, 5 mM MgCl_2_ and 5 mM glucose, pH 6.1) containing 2% (v/v) fetal calf serum and 1% (w/v) bovine serum albumin for 30 min. To differentiate between IMVs and IEVs, cells were stained with a monoclonal antibody against A36 (1:50), kindly provided by Geoffrey Smith (University of Cambridge, UK) (van Eijl et al., 2000), followed by incubation with a Cy5 goat anti-mouse secondary antibody (1:1000; 115-175-146, Jackson ImmunoResearch). To visualise IMVs in ΔB5-infected cells, a monoclonal antibody against A27 (1:1000; Rodriguez et al., 1985) was used followed by a Cy5 goat anti-mouse secondary antibody (1:1000; 115-175-146, Jackson ImmunoResearch). To visualise kinesin-1, the following primary antibodies were used: anti-KIF5B (1:400; ab167429; Abcam), anti-KLC1 (1:400; sc-25735; Santa Cruz) and anti-KLC2 (1:400; HPA040416; Atlas Antibodies); followed by an Alexa Fluor 488 goat anti-rabbit secondary antibody (1:1000; A11034, Invitrogen). Coverslips were mounted on glass slides using Mowiol (Sigma-Aldrich) and images acquired on a Zeiss Axioplan2 microscope equipped with a 63×/1.4 NA Plan-Achromat objective and a Photometrics Cool Snap HQ cooled charge-coupled device camera. The microscope was controlled with MetaMorph 7.8.13.0 software. Images were analysed using Fiji and processed with Adobe software package.

For structured illumination microscopy, fixed samples were prepared as above and imaged on an Olympus iX83 Microscope with an Olympus 150×/1.45 NA X-Line Apochromatic Objective Lens, dual Photometrics BSI-Express sCMOS cameras and CoolLED pE-300 Light Source (Visitech) and was controlled using Micro-Manager 2.0.0. Image stacks of 10–15 *z*-slices with 0.1 µm steps were acquired and deconvolved using the express deconvolution setting on Huygens Software (Scientific Volume Imaging).

For immunoblot analyses, the following antibodies were used: anti-β-tubulin (1:10,000; T7816, Sigma-Aldrich), GFP clone 3E1 (1:5000; Francis Crick Institute, Cell Services STP), anti-KIF5B (1:1000; ab167429; Abcam), anti-KLC1 (1:1000; sc-25735, Santa Cruz Biotechnology), anti-KLC2 (1:1000; HPA040416, Atlas Antibodies), and A27 (1:1000; C3 monoclonal, Rodriguez et al., 1985). HRP-conjugated secondary antibodies were purchased from Jackson ImmunoResearch Laboratories. Original blot images are shown in Fig. S3.

### Live-cell imaging and automated particle tracking in cells

Live-cell imaging experiments were performed at 7.5 h post infection in complete DMEM (10% fetal bovine serum, 1% penicillin/streptomycin) in a temperature-controlled chamber at 37°C. Cells were imaged on a Zeiss Axio Observer microscope equipped with a Plan Achromat 63×/1.40 NA Ph3 M27 oil lens or a Plan Achromat 100×/1.46 NA oil lens, an Evolve 512 camera and a Yokagawa CSUX spinning disc. The microscope was controlled by the SlideBook software (3i Intelligent Imaging Innovations). Time-lapse images used for automated particle tracking were acquired at a sampling rate of 10 Hz using an exposure of 33 ms for the RFP (virus) channel.

To quantify the number of kinesin-1 molecules associated with virions, image stacks of ten *z*-slices that were 0.1 µm apart were acquired at 0.2 Hz using an exposure of 100 ms or 30 ms for the respective GFP (kinesin) and RFP (virus) channels. All other movies were typically imaged at 1 Hz using an exposure of 100 ms for each channel. To visualise microtubules in live cells, 125 nM SiR-tubulin (CY-SC002, Cytoskeleton) was added to the culture medium 2 h prior to imaging.

To track IMVs in infected cells, we used the Fiji plugin, TrackMate (Tinevez et al., 2017). The Laplacian of Gaussian (LoG) detector identified virion spots with an estimated diameter of 1 µm and threshold of 30 using a median filter and sub-pixel localisation. We generated whole-virus trajectories using the simple linear assignment problem (LAP) tracker with a linking distance of 0.8 µm and gap-closing distance of five frames. These were filtered using a track displacement threshold >1 μm. The track data were exported and processed using TraJ (Wagner et al., 2017) to analyse the trajectories, categorising virus tracks into segments (sub-trajectories) representing either active transport, normal diffusion, sub-diffusion or confined motion. The data for active transport were exported to Excel to derive the virion velocities and run lengths. For IEVs, cells infected with WR A36-YdF–YFP RFP–A3 for 7.5 h were imaged on an Olympus iX83 Microscope equipped with an Olympus 150×/1.45 NA X-Line Apochromatic Objective Lens, dual Photometrics BSI-Express sCMOS cameras and CoolLED pE-300 Light Source (Visitech), and was controlled using Micro-Manager 2.0.0. Time-lapse images were acquired at a sampling rate of 10 Hz through simultaneous dual-colour acquisition of the GFP (A36) and RFP (A3) channels using an exposure of 30 ms. IEV tracking was also performed using the TrackMate plugin. The LoG detector identified A3-positive virions with an estimated spot diameter of 0.9 µm and a threshold of 0.4 using the median filter and sub-pixel localisation. Double A3/A36-positive virions were detected by filtering channel 1 A3 spots for simultaneous channel 2 A36 signal detection using a signal-noise ratio filter of 0.4. Generation of IEV trajectories and their subsequent analysis were performed identically to IMVs.

### *In vitro* virus motility assays

HeLa cells were grown in 10 cm culture plates until ∼80% confluency, then infected with the relevant virus for 18 h at a multiplicity of infection of 0.1. Infected cells were detached by versene (prepared in-house by the Crick Media Preparation Team) treatment and centrifuged (580 ***g***, 5 min, 4°C). The cell pellet was resuspended in 0.5 pellet volumes of assay buffer (40 mM HEPES, 1 mM EGTA, 1 mM MgCl_2_, 100 mM KCl, 1% v/v glucose, 1 mM GTP and 10 mM β-mercaptoethanol) supplemented with protease inhibitors (cOmplete Mini EDTA-free, Sigma-Aldrich) and 1 mM phenylmethylsulfonyl fluoride (PMSF). Cells were lysed through two iterative freeze/thaw cycles and the cell lysate was clarified by centrifugation (580 ***g***, 5 min, 4°C). The cell extract was kept on ice for no more than 2 h before the start of imaging. An ATP-regeneration system (2 mM ATP, 25 mM phosphocreatine and 0.013 mg/ml creatine phosphokinase at >150 units/ml) and an oxygen-scavenging system (12.5 mg/ml glucose oxidase and 3 mg/ml catalase) was added to the extract prior to adding into the flow chamber. If polarity-marked microtubules were used, taxol was also added to the extract at a final concentration of 5 µM.

Tubulin was purified from pig brains as previously described (Castoldi and Popov, 2003). Guanylyl-(α,β)-methylene-diphosphonate (GMPCPP)-stabilised microtubules were polymerised from unlabelled tubulin (1.42 µM), biotin-labelled tubulin (0.5 µM) and Alexa Fluor 647-labelled tubulin (0.27 µM) in 1× BRB80 (80 mM K-PIPES pH 6.8, 1 mM MgCl_2_, 1 mM EGTA pH 6.8) containing GMPCPP (0.5 µM) for 3 h at 37°C. Polymerised microtubules were pelleted by centrifugation at 12,420 ***g*** for 5 min, gently resuspended in BRB80 and left in the dark at room temperature overnight for use the following day. Polarity-marked microtubules stabilised with taxol were generated as previously described (Fallesen et al., 2017). In brief, a ‘dim’ tubulin mix containing a low concentration of Alexa Fluor 647-labelled tubulin was polymerised in BRB80 with increasing concentrations of taxol added sequentially: 4 µM, 40 µM and 400 µM taxol for 15 min each at 37°C. A subsequent round of polymerisation was performed by adding a ‘bright’ tubulin mix for a further 15 min at 37°C. The bright tubulin mix contained a high concentration of Alexa Fluor 674-labelled tubulin in addition to N-ethylmaleimide-modified tubulin, which blocks microtubule polymerisation at the minus end. The polymerised microtubules were pelleted by ultracentrifugation using a TLA 120.2 fixed-angle rotor (Beckman Coulter) rotor (96,000 ***g***, 60 min, 35°C) through cushion buffer (60% glycerol v/v in BRB80) supplemented with 20 µM taxol. Finally, the microtubule pellet was washed once and resuspended in BRB80 supplemented with 2 mM dithiothreitol and 20 µM taxol.

Glass coverslips were functionalised with a layer of biotin and biotin-polyethylene glycol (PEG) (Rapp Polymere), whereas glass slides were passivated with poly-L-lysine (PLL)–PEG (SuSoS) as previously described (Bieling et al., 2010). Flow chambers forming a ∼10 µl volume chamber (chamber size ∼0.5×18×0.1 mm), consisted of a biotin–PEG-functionalised coverslip attached to a PLL–PEG-passivated glass slide via double-sided tape (Tesa, Hamburg). The glass surfaces were passivated with 5% Pluronic F-127 (Sigma-Aldrich) followed by κ-casein (0.05 mg/ml) for 10 min each, then incubated with NeutrAvidin (Invitrogen) for 3 min. The polymerised microtubule mix was then added and incubated for 10 min before unattached microtubules were removed by several washes using assay buffer. Finally, the infected cell extract in assay buffer was added and chambers were sealed with Vaseline (Unilever) prior to imaging on a spinning-disc microscope at 37°C. Images were acquired at 1 fps, and each sample was imaged for no longer than 30 min.

### Image analysis and quantitation

Fluorescence intensity measurements of GFP-tagged kinesin-1 or kinesin-1 antibody signal were performed for fixed- or live-cell images using Fiji (Schindelin et al., 2012). Raw integrated kinesin-1 signal was measured following the approach of Verdaasdonk et al. (2014) by drawing an 8-pixel-diameter circle over kinesin-1 spots colocalising with a virion. The background signal was obtained by drawing a larger 10-pixel concentric circle and measuring the raw integrated density. The area-corrected background intensity was subtracted from the initial 8-pixel region of interest to acquire the fluorescence intensity per kinesin-1 spot. Measuring the percentage of IMVs that associate with KIF5B in ΔB5 RFP–A3 infected cells was performed by manually counting the number of virions (A3-positive puncta) outside of the virus factory and wrapping area that were also labelled with the KIF5B antibody using the multi-point tool in Fiji. To quantify the number and percentage of peripheral IMVs in ΔB5 RFP–A3 infections, the cells were stained with phalloidin as a cell mask, and anti-A27 antibody (Rodriguez et al., 1985) to detect IMVs. The outline of the cell was traced using the freehand selection tool and saved as a region of interest (ROI) on Fiji. This ROI was reduced by 5 µm using the enlarge tool to create a smaller ROI. The number of IMVs in each of the ROIs was then determined using the find maxima tool.

The constructs that assembled into 24-, 60- and 120-mer nanocages used for the fluorescence calibration standard curve were generated as described previously (Akamatsu et al., 2020). To generate a construct that would self-assemble into 180-mers, plasmids obtained from the laboratory of David Drubin (University of California, Berkeley, CA) were modified (Akamatsu et al., 2020). The NheI/XbaI fragment was replaced by a synthetic construct (Invitrogen; Geneart) in which the 2-dehydro-3-deoxy-phosphogluconate (KPDG) aldolase was tagged at the N-terminus with two TagGFP2 sequences. GS repeat linkers were included between the TagGFP2 sequences. The nanocage constructs were transiently expressed for ∼26 h in HeLa cells after transfecting cells with Lipofectamine 2000 (Invitrogen) prior to adding 500 nM AP21967 (Takara) to the medium 30 min before imaging to induce self-assembly of nanocages. Average intensity projections of image *z*-stacks were used to measure the fluorescence intensity per nanocage spot. The background-subtracted fluorescence intensity of each nanocage was measured as described above and plotted as a function of predicted TagGFP2 copy number per nanocage to obtain the calibration standard curve. A line of linear fit through the origin was applied by linear least-squares fitting. Identical analysis was performed on TagGFP2–KIF5B spots that colocalised with virions in infected cells to calculate the number of kinesin-1 molecules associated with IMVs or IEVs. Due to the slow (0.2 Hz) rate of imaging *z*-stacks, quantification was only performed on colocalised virion:kinesin puncta that were stationary rather than moving.

To quantify the number of motile IMVs in the nocodazole experiments, TrackMate was used to identify virus trajectories using the parameters described above. A track displacement threshold was applied to all trajectories to quantify the number of viruses that moved >3 µm within the 1 min imaging window. *In vitro* virus motility was analysed by kymograph analysis using the ImageJ Kymograph plugin made by Jens Rietdorf and Arne Seitz (École polytechnique fédérale de Lausanne, Switzerland). The Fiji line tool was used to measure constant velocity segments within kymographs and the data was exported to Excel to derive the virus velocities and run lengths. Virus motility rates were calculated as the total number of motile virions detected, normalised to the imaging duration (in minutes) and sum of all microtubule lengths (in millimetres) within each field of view. The overall virus motility rate per independent experiment is reported.

## Supplementary Material

10.1242/joces.260175_sup1 Supplementary information
