# Supplementary material for: Kinesin-1 transports morphologically distinct intracellular virions during vaccinia infection
Source: J Cell Sci. 2022 Sep 30;136(5):jcs260175. doi: 10.1242/jcs.260175 (PMC9659004; doi:10.1242/jcs.260175)
Supplement: Supplementary information [file joces-136-260175-s1.pdf]

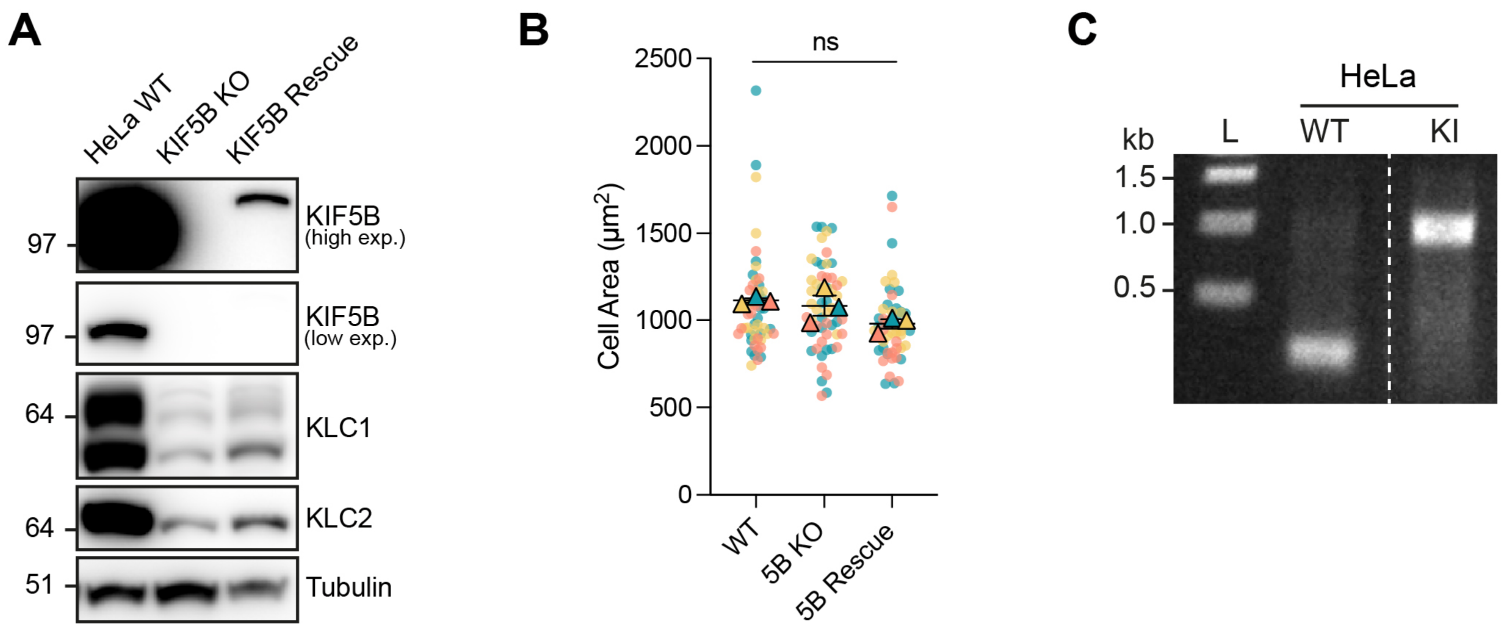

Fig. S1. Validation of TagGFP2-KIF5B HeLa rescue and CRISPR knock-in cell line

(A) Immunoblot analyses with the indicated antibodies of total cell lysates from parental HeLa wild-type (WT), KIF5B knockout (KO) and KIF5B KO stably expressing TagGFP2-KIF5B (termed 'KIF5B Rescue'). The HeLa WT control lane is duplicated and horizontally flipped from Fig. 7D as highlighted in the whole blots in Fig. S3. (B) Quantification of cell area of indicated HeLa cell lines. At least 50 cells were measured from 3 independent experiments. Error bars represent mean and SEM. Ordinary one-way ANOVA test was used to determine statistical significance; ns, $p > 0.05$. (C) Agarose gel electrophoresis showing PCR bands of amplified KIF5B locus in HeLa wild-type (WT) or TagGFP2-KIF5B CRISPR knock-in (KI) cells.

Journal of Cell Science • Supplementary information

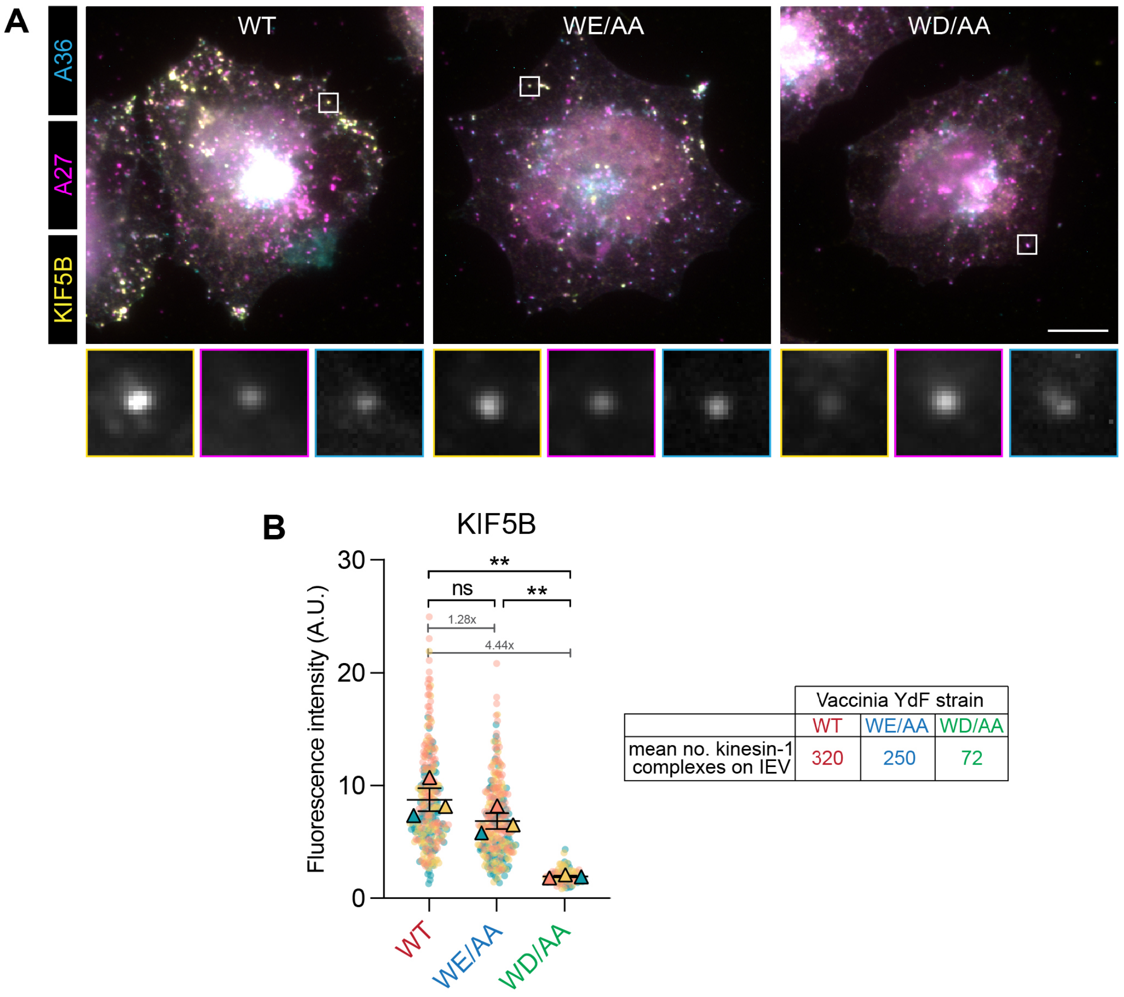

Fig. S2. Quantifying the number of kinesin-1 motors recruited to A36 mutant viruses (A) Representative immunofluorescence images of HeLa cells infected for 8 hours with wildtype (WT) A36-YdF-YFP (blue), or with recombinant strains containing the A36 WE/AA or WO/AA mutations as described previously (Oodding et al., 2011). Cells were also immunolabelled with A27 (magenta) and KIF5B (yellow). Insets show colocalisation of IEV and KIF5B. Scale bar, 10 µm. (B) SuperPlot showing the background subtracted fluorescence intensities of KIF5B associated with IEV produced by the A36-YdF-YFP virus strains in (A). The fold-difference between the means of the WT and mutant strains are indicated. These values were used along with data from Fig. 7H to calculate the mean number of kinesin-1 complexes associated with IEV in each A36-YdF-YFP virus strain as shown in the table (right). Error bars represent mean and SEM from 3 independent experiments. N= 283 (WT), 305 (WE/AA) and 84 (WO/AA) measurements. Tukey's multiple comparison test was used to determine statistical significance; ns, $p > 0.05$, ** $p \leq 0.01$.

Fig. S3. Blot transparency

Related to Fig. 5A:

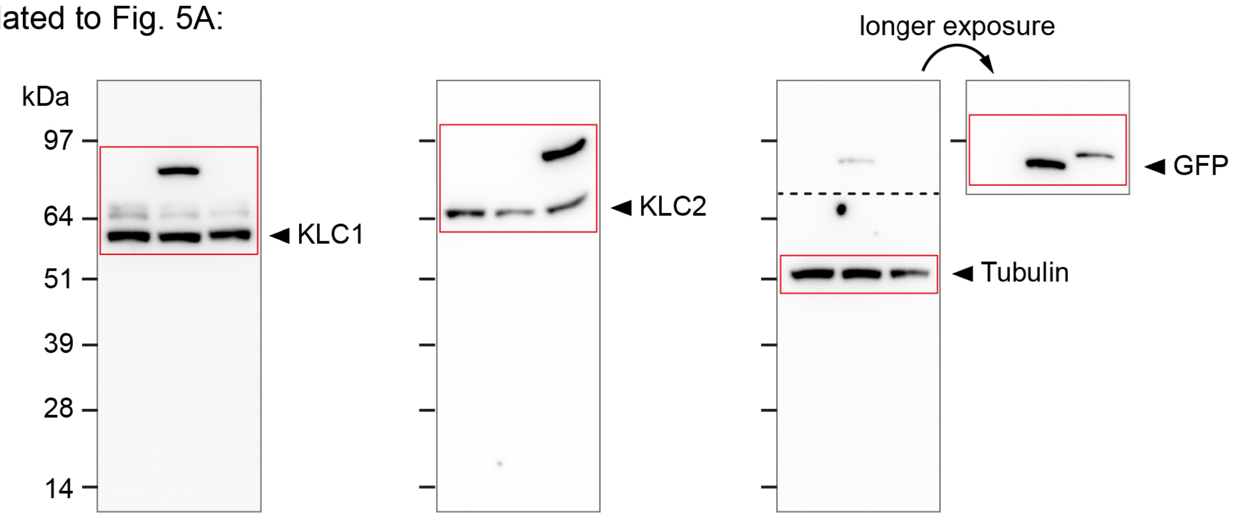

Related to Fig. 5E:

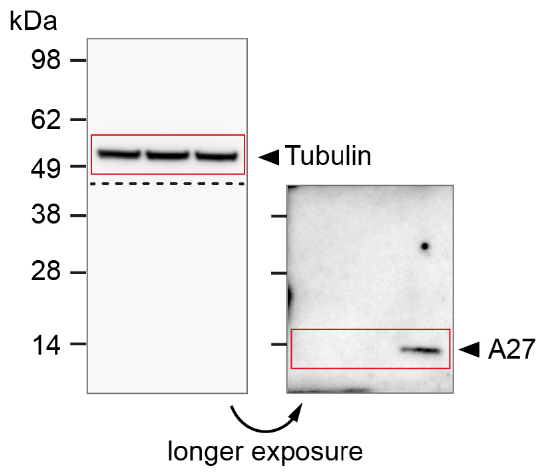

Related to Fig. 7D and Fig. S1:

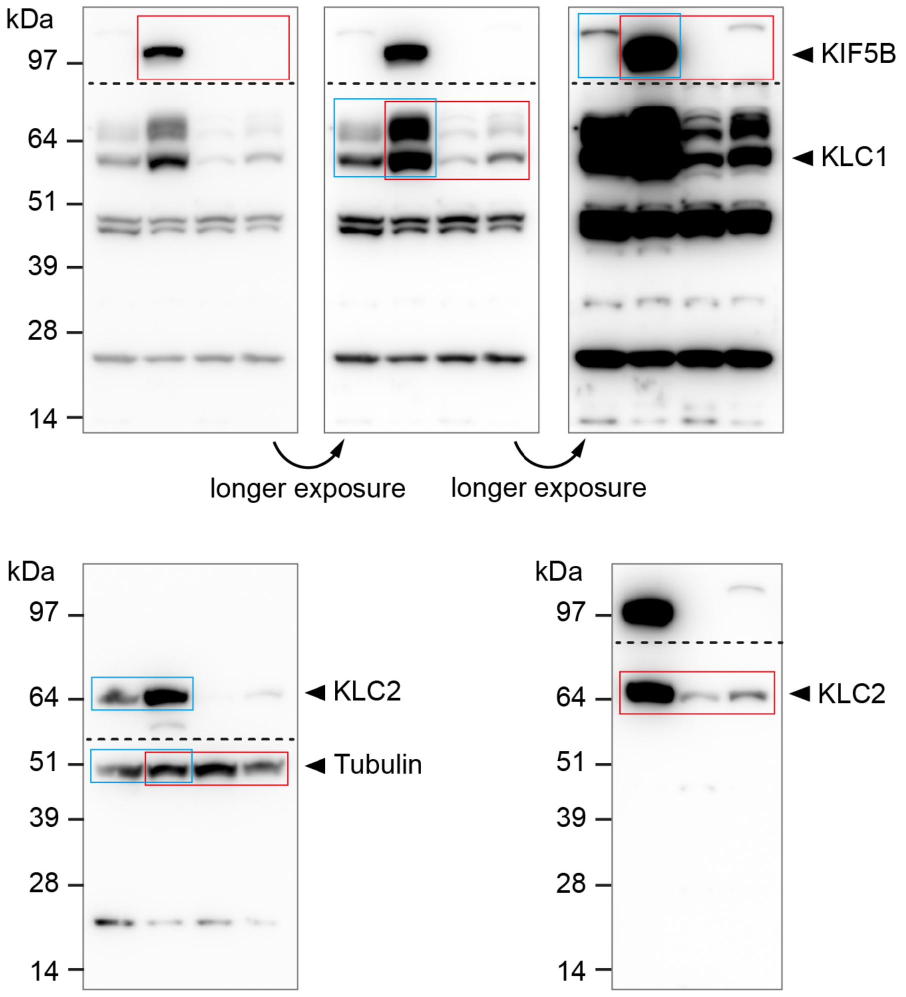

Journal of Cell Science • Supplementary information

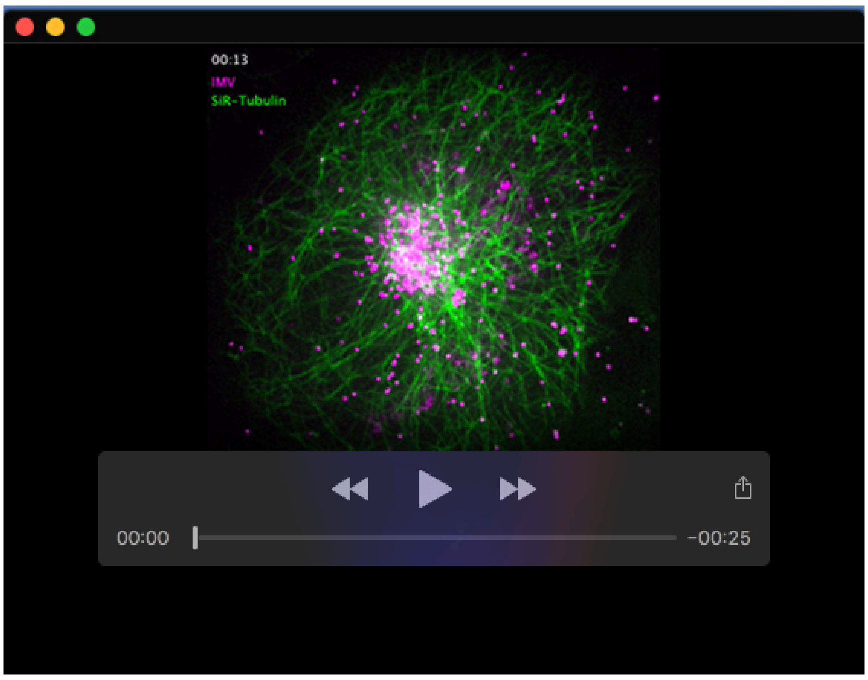

Movie 1. IMV movement in an ΔB5 infected Hela cell (also see Fig. 1A).
Hela cell infected with ΔB5 RFP-A3 for 7.5 h before imaging. The time in minutes:seconds is indicated, and the scale bar= 10 µm. Images were taken every second. Video plays at 10 frames per second.

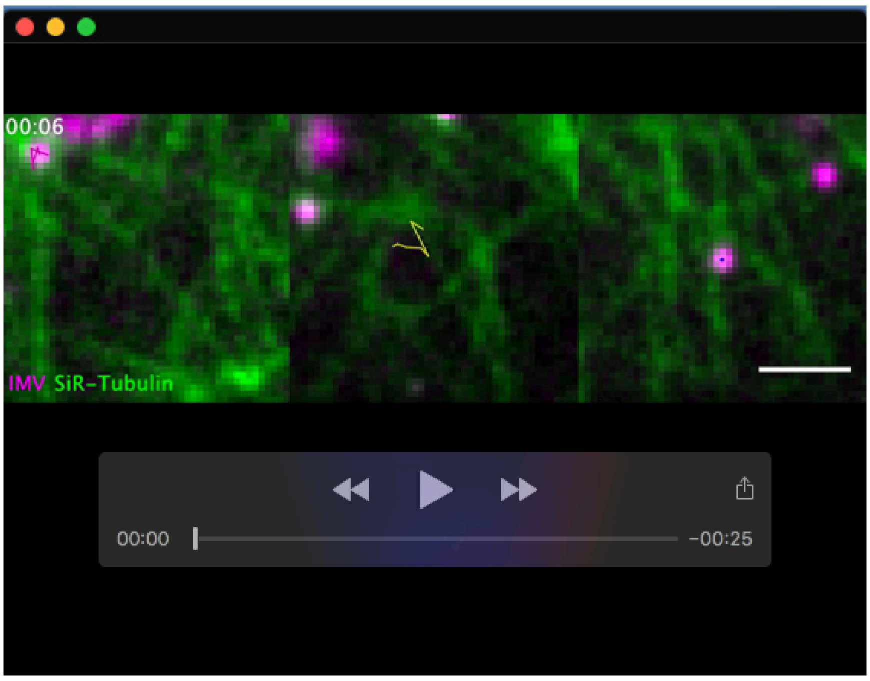

Movie 2. Examples of IMV movements (also see Fig. 1B)
Insets from a Hela cell infected with ΔB5 RFP-A3 for 7.5 h before imaging. Coloured trajectories show active (left), diffusive (middle) and stationary (right) virus movements. The time in minutes:seconds is indicated, and the scale bar = 2 µm. Images were taken every second. Video plays at 10 frames per second.

Journal of Cell Science • Supplementary information

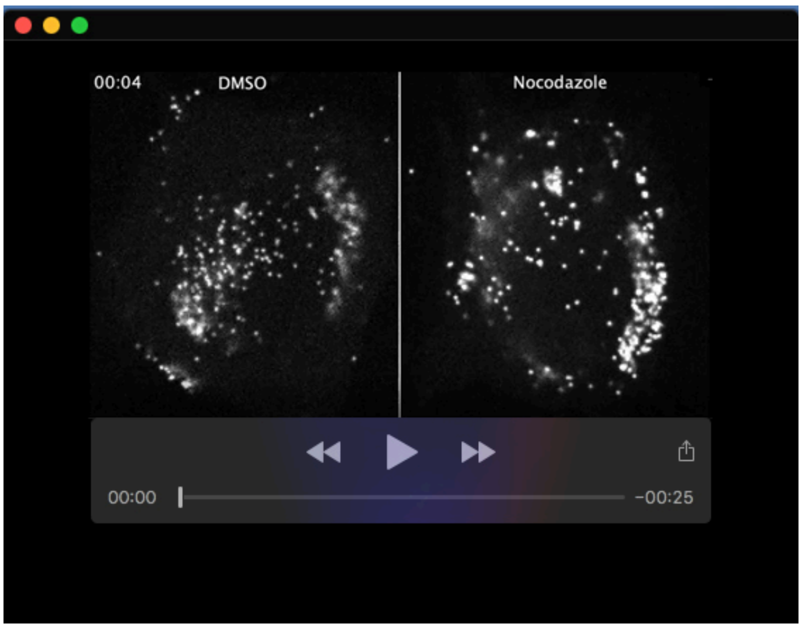

Movie 3. IMV movements in the presence or absence of microtubules (also see Fig. 1D)
Example of a HeLa cell infected with ΔB5 RFP-A3 for 7.5 h and treated with DMSO (left) or 33 μM nocodazole (right) for 1 h before imaging. The time in seconds is indicated, and the scale bar= 10 μm. Images were taken every 0.1 seconds. Video plays at 60 frames per second.

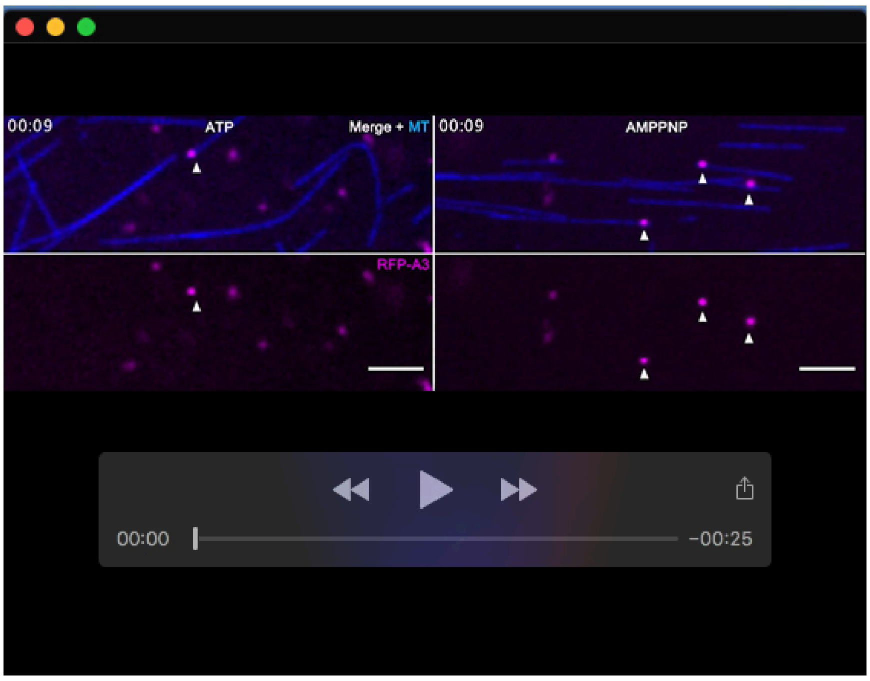

Movie 4. IMV motility on GMPCPP microtubules (also see Fig. 3C)
Example of IMV (magenta) labelled with RFP-A3 (indicated by the white arrowheads) moving on GMPCPP-stabilised microtubules (blue) in vitro in the presence of 2 mM ATP (left panel) but not AMPPNP (right panel). The time in minutes:seconds is indicated, and the scale bar= 5 μm. Images were taken every second. Video plays at 10 frames per second.

Journal of Cell Science • Supplementary information

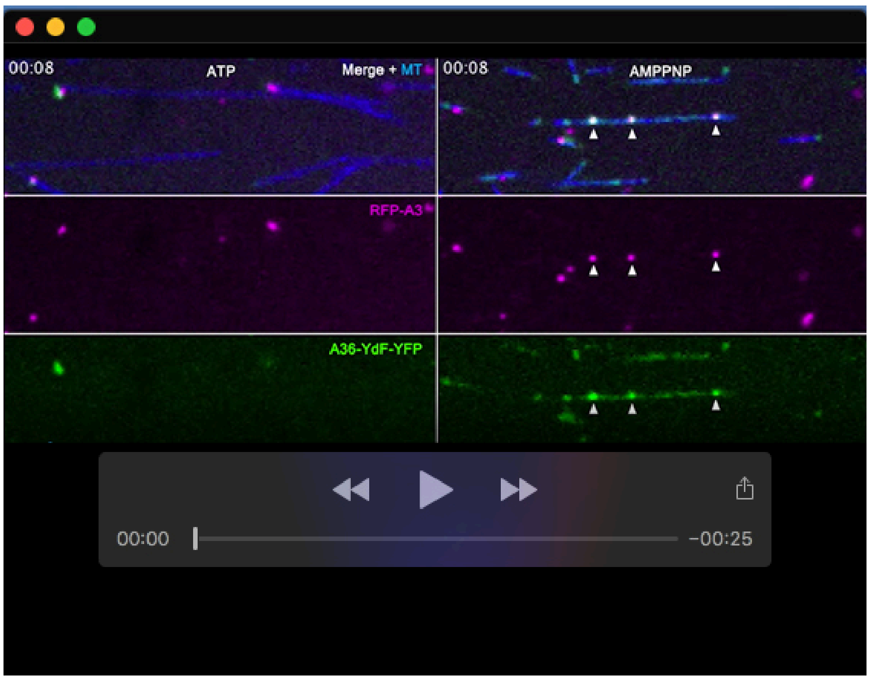

Movie 5. IEV motility on GMPCPP microtubules (also see Fig. 3C)
Example of IEV (magenta) labelled with RFP-A3 and A36-YdF-YFP (green), as indicated by the white arrowheads, moving on GMPCPP-stabilised microtubules (blue) in vitro in the presence of 2 mM ATP (left panel) but notAMPPNP (right panel). The time in minutes:seconds is indicated, and the scale bar= 5 µm. Images were taken every second. Video plays at 10 frames per second.

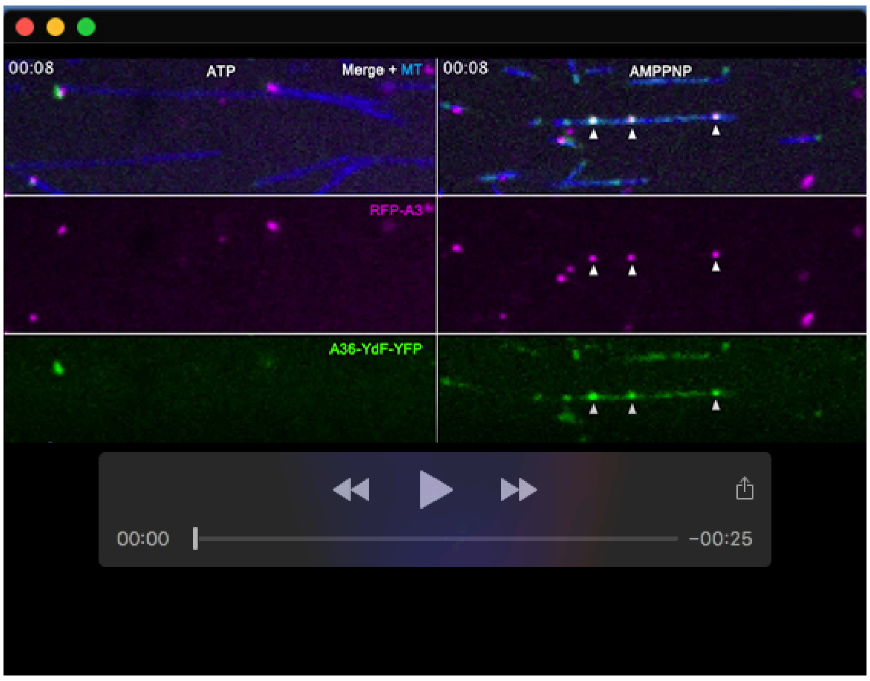

Movie 6. IEV move towards the plus end of microtubules (also see Fig. 3G)
Example of IEV labelled with RFP-A3 (magenta) and A36-YdF-YFP (green), as indicated by the white arrowheads, translocating towards the bright microtubule plus-end (blue) in vitro in the presence of 2 mM ATP. The time in minutes:seconds is indicated, and the scale bar= 10 µm. Images were taken every second. Video plays at 20 frames per second.

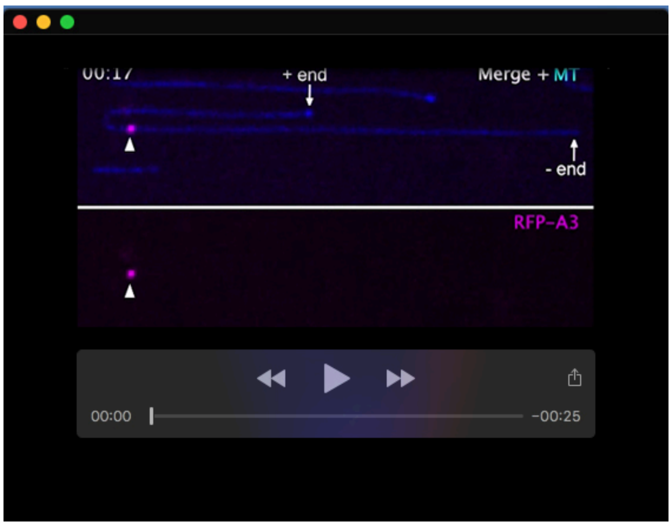

Movie 7. IMV move towards the plus end of microtubules (also see Fig. 3G)
Example of IMV (magenta) labelled with RFP-A3 (indicated by the white arrowheads) translocating towards the bright microtubule plus-end (blue) in vitro in the presence of 2 mM ATP. The time in minutes:seconds is indicated, and the scale bar= 5 μm. Images were taken every second. Video plays at 20 frames per second.

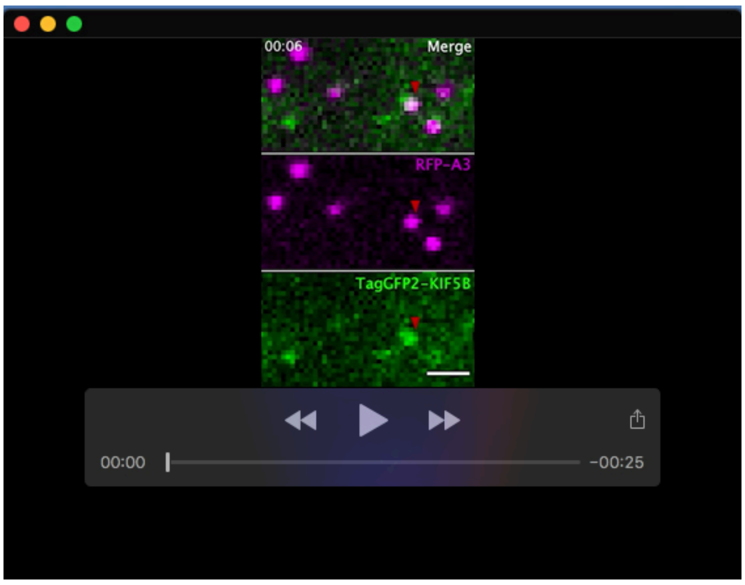

Movie 8. KIF5B associates with moving and stationary IMV (also see Fig. 7E)
Example movie of an IMV labelled with RFP-A3 (magenta), associated with endogenously expressed TagGFP2-KIF5B (green) in Hela knockin cells. The red and blue arrowheads represent periods of IMV movement and stationery. The time in minutes:seconds is indicated, and the scale bar = 2 μm. Images were taken every second. Video plays at 7 frames per second.
