# Supplementary material for: Kinesin-1 transports morphologically distinct intracellular virions during vaccinia infection
Source: J Cell Sci. 2022 Sep 30;136(5):jcs260175. doi: 10.1242/jcs.260175 (PMC9659004; doi:10.1242/jcs.260175)
Supplement: Supplementary file 1 [file joces-136-260175_review_history.pdf]

Kinesin-1 transports morphologically distinct intracellular virions during vaccinia infection

Amadeus Xu, Angika Basant, Sibylle Schleich, Timothy P. Newsome and Michael Way
DOI: 10.1242/jcs.260175

Editor: Anne Straube

Review timeline
Original submission: 27 April 2022
Editorial decision: 30 May 2022
First revision received: 23 August 2022
Accepted: 31 August 2022

Original submission

First decision letter

MS ID#: JOCES/2022/260175

MS TITLE: Kinesin-1 transports morphologically distinct intracellular virions during vaccinia infection

AUTHORS: Amadeus Xu, Angika Basant, Sibylle Schleich, Timothy P Newsome, and Michael Way
ARTICLE TYPE: Research Article

We have now reached a decision on the above manuscript.

To see the reviewers' reports and a copy of this decision letter, please go to: https://submit-jcs.biologists.org and click on the 'Manuscripts with Decisions' queue in the Author Area. (Corresponding author only has access to reviews.)

As you will see, the reviewers gave favourable reports but raised some critical points that will require amendments to your manuscript. I hope that you will be able to carry these out because I would like to be able to accept your paper, depending on further comments from reviewers.

Please ensure that you clearly highlight all changes made in the revised manuscript. Please avoid using 'Tracked changes' in Word files as these are lost in PDF conversion.

I should be grateful if you would also provide a point-by-point response detailing how you have dealt with the points raised by the reviewers in the 'Response to Reviewers' box. Please attend to all of the reviewers' comments. If you do not agree with any of their criticisms or suggestions please explain clearly why this is so.

Reviewer 1

Advance summary and potential significance to field

In this manuscript, Xu and colleagues examine the role of kinesin in regulating the motility of the IMV form of progeny poxvirus virions, which is more abundant but less well understood in terms of intracellular transport than the IEV form. The subject matter is of interest and importance to both the poxvirus and microtubule motor field. The experiments are well designed and executed. For

© 2022. Published by The Company of Biologists under the terms of the Creative Commons Attribution License (https://creativecommons.org/licenses/by/4.0/).

this reason, I have only relatively minor comments that are aimed at addressing conceptual curiosities that might be worth discussing or clarifying for a general audience.

Comments for the author

IMV moves slower than IEV in cells but this phenomenon seems to be reversed in vitro. What do the authors think is causing this? Could it be that microtubules in cells carry PTMs that affect kinesin activity that are not present on in vitro filaments? This doesn't impact the importance of the results, it may just be worth commenting on.

Perhaps related to this, tagGFP2-Kif5B seems to robustly rescue in cells but only partially rescues in cell extracts and in vitro approaches. Might this also relate to PTMs? Alternatively, does tagGFP2-Kif5B tend to localize more peripherally in cells and thereby function more efficiently in rescue? What is the localization pattern of tagGFP2-Kif5B in uninfected cells? The degree of rescue is surprising given the relatively low expression level, but this might relate to localization or the point below – namely, are many IMV not fully matured and primed for motor engagement, and therefore there is not a lot of competition for the small amount of available rescue kinesin?

The imaging clearly shows that a large amount of IMV in particular do not colocalize with kinesin. I am guessing these are IMV that are not fully matured or competent to engage kinesin yet. This lack of kinesin engagement likely explains why a large population IMV is not motile, and seems like a simple but important observation in itself.

If a cargo only requires 3 kinesin motors to engage a microtubule filament, why would IMV be slower than IEV as they both engage a large number of kinesins. Do the motors "burn out" and switch on cargos, and that underlies why IEV is faster?

Movements are largely unidirectional. Is it known whether IMV or IEV engage dynein or purely kinesin to favor anterograde movement predominates?

Are KCL1/2 reduced in knockout cells because Kif5B is required for their stability?

Page 4, middle of first paragraph. The authors mention diffusion within the MT network. In the movies it seems they circle within a "cage" of criss-crossed MTs that surrounds them. Could some of what seems like diffusive movement actually just be particles switching from one filament to another when located in these MT junctions? Do large IMV diffuse efficiently? Perhaps what has been reported as diffusion is actually that the authors have imaged?

Page 3, end of the first paragraph. I'm not quite sure what's meant by "suggesting the virus also regulates motor recruitment" as it is discussing a second viral protein, after another protein that also recruits the motor has already been discussed and it is established that recruitment is regulated. Do the authors mean that it is recruited in a complex manner involving multiple proteins?

What was used to polarity-mark the MT ends? A paper is cited but it would be helpful to know the marker used.

<u>Reviewer 2</u>

Advance summary and potential significance to field

This manuscript builds on previous work from the Way lab investigating the role of kinesins and microtubules in the intracellular trafficking and maturation of vaccinia virus. Previous work investigated the first stage in vaccina virus production, the assembly of intracellular mature virions (IMVs), and found that kinesin-1 is required for transport to the TGN. This manuscript is focused on the second stage in vaccina virus production, the assembly of intracellular enveloped virions (IEVs). Using viral strains that produce both IMVs and IEVs or just IMVs (strain deltaB5) and quantitative microscopy, the authors demonstrate that motility of IMVs and IEVs can be reconstituted on microtubules in vitro in an ATP-dependent manner. They also show that in cells, transport of IEVs

© 2022. Published by The Company of Biologists under the terms of the Creative Commons Attribution License (https://creativecommons.org/licenses/by/4.0/).

and IMVs is dependent on the kinesin-1 motor protein KIF5B+KLC1/2 and that IEV recruit more kinesin-1 molecules than IMV. The work is important, the data and analysis are of high quality and integrity, and the manuscript is well-written.

Comments for the author

Using nanocages to generate a standard curve for quantifying numbers of molecules on a cargo is novel and exciting. However, the authors come to the surprising conclusion that there are hundreds of kinesins on a IMV or IEV cargo which is in contrast to previous work suggesting that membrane-bound cargoes have only a handful of kinesins. The images in Figure 4E appear to corroborate this as the KIF5B spots appear to completely colocalize with the viral proteins and not other spots. Can the authors measure the % colocalization? If true, then it would seem that the virus is much better than any cellular cargo at recruiting kinesin-1.
Given the newness of the technique and its use in uncovering one of the highlights of the manuscript, it seems important to verify the approach. The size of the nancages and IMV and IEV seem fairly similar. Are they and is this surprising? Is it possible that there is fluorescence quenching within the confines of the nanocage which would result in lower than expected fluorescence intensities? Do the authors get the same results when immunostaining for kinesin-1 as they do for the TagGFP2 protein? Does the amount of kinesin-TgGFP2 vary depending on localization within the cell (e.g. near nucleus vs at periphery) or time of infection?

Reviewer 3

Advance summary and potential significance to field

While it is known that the vaccinia virus (VV) protein A36 of intracellular enveloped virions (IEV) recruits kinesin-1 and mediates transport to the plasma membrane, the microtubule motors for motility of the intracellular mature virions (IMV) are less characterized.
To investigate IMV motility, Xu et al. used the mutant VV-deltaB5-RFP-A3, which expresses an RFP-tagged core protein A3 and assembles infectious IMVs but no IEVs for characterizing the velocity and the run length of various VV particles in vivo and in vitro. Towards this end, the authors established a novel biochemical VV transport assay using polarity marked microtubules. The development of this in vitro assay is a major achievement towards future research on VV motility. In cells, IMV and IEV co-localized with the kinesin-1 heavy chain KIF5B and the light chains KLC1 and KLC2, although the kinesin-1 signals on IMVs were lower than on IEVs. Using the novel in vitro assay, the authors showed unidirectional transport towards the microtubule plus-ends for both, IMV and IEV, which could be blocked by the kinesin inhibitor AMPPNP. Another mutant, VV-deltaA27 moved with similar velocities and run lengths indicating that the IMV protein A27 did not contribute to the recruitment of kinesin-1.
Kinesin-1 was required for peripheral accumulation of IMV and IEV as shown in KIF5B rescue cells bot not in KIF5B KO cells, and extracts from KIF5B rescue cells bot not from KIF5B KO cells promoted VV motility in vitro. Furthermore the C-terminal TPR domain of KLC1 or KLC2 was required and sufficient for recruitment to IMV. The authors estimated that IMV recruited 65 and IEV 115 kinesin-1 complexes by comparing the amounts of associated TagGFP2-tagged KIF5B with calibrated TagGFP2 nanocages.
This study delivers new insights on motor usage of VV particles which are of interest to both, virologists but also cell biologists, as viruses serve as important model cargoes for microtubule transport. Overall, the figures support the conclusions, and the manuscript is written very clearly and compact.

Comments for the author

1. Introduction – discussion: I am missing a reference to Schepis et al. 2007 (Cellular Microbiology 9:1960) who also reported on the role of kinesin-1 and possibly kinesin-2 for transport of IMV and IEV to the plasma membrane. Please integrate and discuss in light of the data present in this manuscript.
2. The author made a strong point regarding the unidirectional in vitro motility of IMV and IEV towards the microtubule plus-ends. However, these data do not exclude the possibility that IMV and IEV might be able to also recruit the minus-end directed motor dynein. The set-up of their in vitro

© 2022. Published by The Company of Biologists under the terms of the Creative Commons Attribution License (https://creativecommons.org/licenses/by/4.0/).

reconstitution might simply not have supported dynein activity. Please explain and modify the discussion accordingly.
3. As the expression of knock/in TagGFP2-KIF5B was low compared to endogenous KIF5B expression, it is likely that this lead to a considerable underestimation of the number of IMV or IEV associated kinesins. Any idea why the expression of TagGFP2-KIF5B is so weak? As I understood, the authors did select for cells with biallelic expression. Please explain and modify accordingly.

Minor comments:
1. Please add a few sentences explaining the concept of using the nanocages.
2. Some abbreviations are not defined: e.g., GMPCPP, BME, PEG, PLL, and KPDG.
3. Page 9, line 16: “long” appears to be superfluous 4. Page 10, line 6: missing bracket;
5. Page 13, last paragraph, line 3: 1 % P/S: remove empty space;
6. Page 14, lines 4-6: check syntax;
7. Page 14, line 21: do not capitalize “Glucose”
8. Page 14, line 27: do not capitalize “Units”
9. Page 14, line 32: change “MgCl2” to have 2 as a subscript.

First revision

<u>Author response to reviewers' comments</u>

We thank the reviewers’ for their comments and suggestions which have definitely improved the paper and uncovered that we were under estimating the number of kinesin-1 motors associated with virions.

Reviewer 1 Advance Summary and Potential Significance to Field:

In this manuscript, Xu and colleagues examine the role of kinesin in regulating the motility of the IMV form of progeny poxvirus virions, which is more abundant but less well understood in terms of intracellular transport than the IEV form. The subject matter is of interest and importance to both the poxvirus and microtubule motor field. The experiments are well designed and executed. For this reason, I have only relatively minor comments that are aimed at addressing conceptual curiosities that might be worth discussing or clarifying for a general audience.

Reviewer 1 Comments for the Author:

IMV moves slower than IEV in cells but this phenomenon seems to be reversed in vitro. What do the authors think is causing this?

Previous analysis of IEV speeds in Dodding et al 2011 used manual tracking rather than the automated method we used for IMV. To determine whether the difference is real or due to the method that was used to track the virions we have now also analysed IEV movements using our automated approach. This data, which is now presented in revised Figure 2, shows that IEV movements in cells and in vitro are identical (0.56 ± 0.28 and 0.56 ± 0.08 μm/s respectively). These values are more similar to IMV (0.61 ± 0.35 μm/s in cells and 0.66 ± 0.14 μm/s in vitro) than the manually tracked IEV in Dodding et al 2011 (0.88 ± 0.04 μm/s). The larger number of automated tracked IEV (3518 compared to 65) also gives us more confidence in the values we obtained so we think it is clear that the discrepancy arose from the tracking method.

Could it be that microtubules in cells carry PTMs that affect kinesin activity that are not present on in vitro filaments? This doesn’t impact the importance of the results, it may just be worth commenting on.

It’s possible that there are differences in PTMs between the MTs in cells vs in vitro given their source, which could influence kinesin activity differently between the two systems. However,

© 2022. Published by The Company of Biologists under the terms of the Creative Commons Attribution License (https://creativecommons.org/licenses/by/4.0/).

given our new data on IEV movement in cells and the close alignment between cellular and in vitro speeds for IMV and IEV we do not think that PTMs are having a major impact on virion movements – possibly because of the large number of associated motors.

Perhaps related to this, tagGFP2-Kif5B seems to robustly rescue in cells but only partially rescues in cell extracts and in vitro approaches. Might this also relate to PTMs?

We cannot be sure, but we think the most likely explanation is due to differences in tagGFP2-Kif5B concentration (which is already lower than Kif5B in the parental WT cells). In the in vitro assay the cell extract is diluted ~ 2 fold, which will result in a further reduction in the concentration of kinesin-1 in the system compared to the rescue cells. This notion is also consistent with the observation that in vitro the rescue for IEV, which are more efficient at recruiting kinesin-1, is better than IMV.

Alternatively, does tagGFP2-Kif5B tend to localize more peripherally in cells and thereby function more efficiently in rescue? What is the localization pattern of tagGFP2-Kif5B in uninfected cells?

In our widefield microscope the TagGFP2-KIF5B localisation in uninfected rescue cells is largely diffuse and cytoplasmic, with higher signal towards the perinuclear region in some cells. We also see the signal associated with moving puncta in live cells, which are presumably vesicles.

The degree of rescue is surprising given the relatively low expression level, but this might relate to localization or the point below – namely, are many IMV not fully matured and primed for motor engagement, and therefore there is not a lot of competition for the small amount of available rescue kinesin? The imaging clearly shows that a large amount of IMV in particular do not colocalize with kinesin. I am guessing these are IMV that are not fully matured or competent to engage kinesin yet. This lack of kinesin engagement likely explains why a large population IMV is not motile, and seems like a simple but important observation in itself.

Our IF analysis on fixed images reveals that only a small proportion 4.6 % of IMV recruit kinesin-1 at any given time which explains why most IMV are not motile (this information is now in the results on page 5). What is not immediately obvious is why the majority of IMV are not motile, as all IMV in the cytoplasm (outside of their perinuclear site of assembly) are morphologically mature (based on EM images in many studies). This also holds true in ΔB5 RFP-A3 infected cells so the lack of motor recruitment is not because of competition for the motor with IEV. We assume that they are not competent to recruit kinesin-1 presumably because they lack some modification. We agree it is an important observation and have now mentioned this in the discussion.

If a cargo only requires 3 kinesin motors to engage a microtubule filament, why would IMV be slower than IEV as they both engage a large number of kinesins. Do the motors “burn out” and switch on cargos, and that underlies why IEV is faster?

Our new analysis reveals IMV are in fact marginally faster than IEV both in vitro and in cells. It is possible that this small difference reflects the fact that IEV are twice the volume of IMV (See Hernandez-Gonzalez et al., 2022). IEV, however, have higher motility rate (in vitro) and longer run lengths (in cells), which is consistent with them recruiting more motors. In addition, the motor density is greater on IEV than IMV (1 motor complex per 1265 nm^2 for IEV as compared to 1715 nm^2 for IMV).

It is impossible to know if motors burn out on virions, but we do see that not all virions make it to the ends of microtubules in our in vitro motility assays (Fig. 3E). We would assume that motors can switch (its not the same motors engaged with the MT from start to finish) and having a higher number and/or density helps ensure the virion remains associated with the MT. The dynamics and activity of motors on cargoes is an interesting area for future research and Vaccinia might provide a good model to tackle these interesting questions.

Movements are largely unidirectional. Is it known whether IMV or IEV engage dynein or purely kinesin to favor anterograde movement predominates?

© 2022. Published by The Company of Biologists under the terms of the Creative Commons Attribution License (https://creativecommons.org/licenses/by/4.0/).

We spent many years trying to detect dynein on virions with antibodies and using a DHC-GFP cell line with no success. However, in the literature, visualising dynein on any cargo is generally very challenging. It is possible that virions recruit a small number of dyneins which are below the threshold of detection using fluorescence-based imaging methods. However, in our in vitro assays we don't see any minus plus end directed transport even in the absence of kinesin-1, so we favour dynein is not there.

Are KCL1/2 reduced in knockout cells because Kif5B is required for their stability?

Based on our blots (see Fig. S1A) it looks as if KLC1/2 levels are lower in the absence of Kif5B. However, looking through the literature we found another paper reporting KO of Kif5B in in RPE1 cells (Robert et al., 2019 FASEB Jan;33(1):388-399. PMID: 29944446 doi: 10.1096/fj.201800604R - Kinesin-dependent transport of keratin filaments: a unified mechanism for intermediate filament transport). The western blot in Figure 4D clearly shows that loss of Kif5B leads to a dramatic reduction in KLC1 and KLC2. The same study also shows that KO of KLC1 does not impact levels of KIF5B presumably as KLC2 is still present. Based on our observations and this study, it looks highly likely that KLC1/2 stability is dependent on Kif5B.

Page 4, middle of first paragraph. The authors mention diffusion within the MT network. In the movies it seems they circle within a "cage" of criss-crossed MTs that surrounds them. Could some of what seems like diffusive movement actually just be particles switching from one filament to another when located in these MT junctions? Do large IMV diffuse efficiently? Perhaps what has been reported as diffusion is actually that the authors have imaged?

In movie 2, the virion in question seems to move randomly within the MT "cage" and there is no sign that it remains attached to any microtubule and stationary for any extended time. Given this we favour diffusion, as we have observed other examples of similar motions. It has been calculated that it would take 5-6 hours for newly assembled virions to diffuse 10 µm (Sodeik, 2000).

Page 3, end of the first paragraph. I'm not quite sure what's meant by "suggesting the virus also regulates motor recruitment" as it is discussing a second viral protein, after another protein that also recruits the motor has already been discussed and it is established that recruitment is regulated. Do the authors mean that it is recruited in a complex manner involving multiple proteins?

We mean to say that recruitment of kinesin-1 to A36 may be regulated by an additional cytosolic viral protein complex such as E2/F12, given it associates with moving IEV. Presence of E2/F12 has also been suggested to enhance binding of kinesin to A36, based on CoIPs (Carpentier et al., 2015; Gao et al., 2017).

What was used to polarity-mark the MT ends? A paper is cited but it would be helpful to know the marker used.

A protein marker is not used. Rather, the MTs are polymerised in 2 sequential rounds. In the first round, a low concentration of AF647-labelled tubulin is used. This is followed by a second, shorter round of polymerisation using a high concentration of AF647-labelled tubulin, as well as NEM-labelled tubulin, which prevents MT growth at the minus end.

Reviewer 2 Advance Summary and Potential Significance to Field:

This manuscript builds on previous work from the Way lab investigating the role of kinesins and microtubules in the intracellular trafficking and maturation of vaccinia virus. Previous work investigated the first stage in vaccina virus production, the assembly of intracellular mature virions (IMVs), and found that kinesin-1 is required for transport to the TGN. This manuscript is focused on the second stage in vaccina virus production, the assembly of intracellular enveloped virions (IEVs). Using viral strains that produce both IMVs and IEVs or just IMVs (strain deltaB5) and quantitative microscopy, the authors demonstrate that motility of IMVs and IEVs can be reconstituted on microtubules in vitro in an ATP-dependent manner. They also show that in cells, transport of IEVs and IMVs is dependent on the kinesin-1 motor protein KIF5B+KLC1/2 and that IEV

© 2022. Published by The Company of Biologists under the terms of the Creative Commons Attribution License (https://creativecommons.org/licenses/by/4.0/).

recruit more kinesin-1 molecules than IMV. The work is important, the data and analysis are of high quality and integrity, and the manuscript is well-written.

Reviewer 2 Comments for the Author:

Using nanocages to generate a standard curve for quantifying numbers of molecules on a cargo is novel and exciting. However, the authors come to the surprising conclusion that there are hundreds of kinesins on a IMV or IEV cargo which is in contrast to previous work suggesting that membrane-bound cargoes have only a handful of kinesins. The images in Figure 4E appear to corroborate this as the KIF5B spots appear to completely colocalize with the viral proteins and not other spots. Can the authors measure the % colocalization? If true, then it would seem that the virus is much better than any cellular cargo at recruiting kinesin-1.

To address the reviewers question we have quantified the % co-localisation of virus and tagGFP-KIF5B puncta from the IF images of 10 cells that were used to generate the data in Fig 6E (same as original Fig. 4E). We find that of the KIF5B puncta, 90.1% are A3 positive and 9.9% lack the viral marker. This suggests that the virus is very efficient at recruiting KIF5B. We have not added this result to the paper as we feel it is hard to make comparison to non-infected cells and we also have no information concerning the identity of the non-viral cargoes and whether they also compete for KIF5B in non-infected cells.

Given the newness of the technique and its use in uncovering one of the highlights of the manuscript, it seems important to verify the approach. The size of the nanocages and IMV and IEV seem fairly similar. Are they and is this surprising?

The method we are using has previously been published by the Drubin lab Akamatsu et al. (2020). The nanocage is ~ 25 nm in diameter (Hsia et al., 2016 Design of a hyperstable 60-subunit protein icosahedron. Nature 535, 136– 139. https://doi.org/10.1038/nature18010), whereas a virion is considerably bigger: IMV 350 x 280 x 200 nm and IEV 440 x 380 x 260 nm based on our cryo-EM of infected cells (see Hernandez-Gonzalez et al., 2022). Both appear a similar size because we do not have enough resolving power in our microscope because - each pixel on our Evolve camera equates to 133 nm. The figure below shows example line scans of the GFP signal from 10 nanocages or virions.

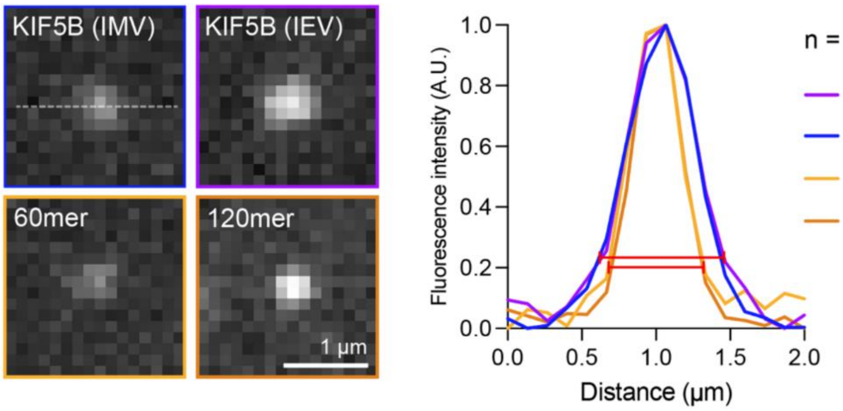

Is it possible that there is fluorescence quenching within the confines of the nanocage which would result in lower than expected fluorescence intensities?

We cannot rule out that there is some quenching. However, the calibration curves in Akamatsu et al. (2020) and the original paper from the Baker lab who designed the cages (Hsia et al., 2016 Design of a hyperstable 60-subunit protein icosahedron. Nature 535, 136-139. https://doi.org/10.1038/nature18010) as well as our data (Fig. 7G) are linear. This would suggest that any quenching is minimal as the different cages would not all be expected to behave the same given the arrangement of GFP molecules inside and on surface of the nanocages varies. The 24mer has 12 inside the cage and 12 outside; the 60mer only has 60 inside; the 120mer has 60 inside the cage and 60 outside and the 180mer has 60 inside the cage and 120 outside.

© 2022. Published by The Company of Biologists under the terms of the Creative Commons Attribution License (https://creativecommons.org/licenses/by/4.0/).

Do the authors get the same results when immunostaining for kinesin-1 as they do for the TagGFP2 protein?

Using a KIF5B antibody we found that IEV recruit 2.66 fold more kinesin-1 than IMV (Fig. 4B), whereas using the TagGFP2-KIF5B knockin cell lines the difference was 1.77 fold. This promoted us to examine the levels of kinesin- 1 on IMV and IEV in the parental and TagGFP2-KIF5B knockin cell lines using the KIF5B antibody to see if we got the same values. We found that IMV and IEV recruited 2.14 and 2.78 fold more kinesin in the parental cells compared to the knockin line (Fig. 7H). We believe this difference reflects the lower expression levels of TagGFP2- KIF5B compared to the untagged KIF5B in the parental cells. Importantly, this analysis clearly indicated that we were underestimating the number kinesin-1 motors on IMV and IEV. The ratio of the corrected values (139 for IMV and 320 for IEV) is now more similar to the fold difference in Fig. 4B (2.30 vs 2.66).

Does the amount of kinesin-TgGFP2 vary depending on localization within the cell (e.g. near nucleus vs at periphery) or time of infection?

In uninfected cells, tgGFP2-KIF5B is largely cytoplasmic and diffuse while in some cells, there is some accumulation at the perinuclear region. During infection, localisation at the perinuclear region remains, but there is additional accumulation of kinesin at the cell periphery/tips, as well as on virions which recruit kinesin (most noticeable for IEV).

Reviewer 3 Advance Summary and Potential Significance to Field:

While it is known that the vaccinia virus (VV) protein A36 of intracellular enveloped virions (IEV) recruits kinesin-1 and mediates transport to the plasma membrane, the microtubule motors for motility of the intracellular mature virions (IMV) are less characterized. To investigate IMV motility, Xu et al. used the mutant VV-deltaB5-RFP-A3, which expresses an RFP-tagged core protein A3 and assembles infectious IMVs but no IEVs for characterizing the velocity and the run length of various VV particles in vivo and in vitro. Towards this end, the authors established a novel biochemical VV transport assay using polarity marked microtubules. The development of this in vitro assay is a major achievement towards future research on VV motility. In cells, IMV and IEV co-localized with the kinesin- 1 heavy chain KIF5B and the light chains KLC1 and KLC2, although the kinesin-1 signals on IMVs were lower than on IEVs. Using the novel in vitro assay, the authors showed unidirectional transport towards the microtubule plus- ends for both, IMV and IEV, which could be blocked by the kinesin inhibitor AMPPNP. Another mutant, VV-deltaA27 moved with similar velocities and run lengths indicating that the IMV protein A27 did not contribute to the recruitment of kinesin-1. Kinesin-1 was required for peripheral accumulation of IMV and IEV as shown in KIF5B rescue cells bot not in KIF5B KO cells, and extracts from KIF5B rescue cells bot not from KIF5B KO cells promoted VV motility in vitro. Furthermore, the C-terminal TPR domain of KLC1 or KLC2 was required and sufficient for recruitment to IMV. The authors estimated that IMV recruited 65 and IEV 115 kinesin-1 complexes by comparing the amounts of associated TagGFP2-tagged KIF5B with calibrated TagGFP2 nanocages.This study delivers new insights on motor usage of VV particles which are of interest to both, virologists but also cell biologists, as viruses serve as important model cargoes for microtubule transport. Overall, the figures support the conclusions, and the manuscript is written very clearly and compact.

Reviewer 3 Comments for the Author:

1. Introduction – discussion: I am missing a reference to Schepis et al. 2007 (Cellular Microbiology 9:1960) who also reported on the role of kinesin-1 and possibly kinesin-2 for transport of IMV and IEV to the plasma membrane. Please integrate and discuss in light of the data present in this manuscript.

Schepis use a Kin330-GFP construct consisting of first 330 amino acids (i.e. the motor domain of kinesin-1) fused to GFP, which appears to work as a dominant negative by a mechanism that is not immediately obvious (see authors discussion). No data is presented showing Kin330-GFP is recruited to virions.

© 2022. Published by The Company of Biologists under the terms of the Creative Commons Attribution License (https://creativecommons.org/licenses/by/4.0/).

Overexpression of this construct in infected cells led to a reduction in the area occupied by virions detected with p14/A27 antibody labelling (A27 cell area coverage: ~50% in GFP control vs ~30% in Kin330-GFP). They also found that % of cells with actin tails, which only form at the plasma membrane after IEV transport on MTs is also reduced (GFP control: ~40%, Kin330-GFP: ~15%).

The main issue is that in this study it is not clear whether the authors are looking at IMV or IEV as the p14 antibody which detects A27 would label both forms. Thus, it is impossible to conclude based on area occupied by p14 positive virions whether Kin330-GFP inhibits transport IMV, IEV or both. In contrast, the reduction in actin tails would point to an inhibition in IEV transport although in the absence of an IEV marker it is possible that Kin330- GFP impacts IEV formation by an indirect mechanism. Given these issues, we have only added the reference in the first paragraph of the discussion (page 9) but have not discussed the paper further as we think there are multiple explanations for the observations. There is also no evidence in the paper supports a role for kinesin-2 in the movement of IMV or IEV.

2. The author made a strong point regarding the unidirectional in vitro motility of IMV and IEV towards the microtubule plus-ends. However, these data do not exclude the possibility that IMV and IEV might be able to also recruit the minus-end directed motor dynein. The set-up of their in vitro reconstitution might simply not have supported dynein activity. Please explain and modify the discussion accordingly.

This is true, we have not tested dynein functionality in our in vitro assays and cannot firmly exclude a possible role for dynein in virion motility. Over the years, we have never managed to detect dynein on IMV or IEV using multiple antibodies or GFP-tagged proteins in live or fixed cells. We also find that IMV move to the plus end of MTs in the absence of kinesin-1 in our in vitro assays. We cannot formally rule out that IMV and /or IEV do not recruit a handful of dynein motors that are below our level of detection. In the absence of any evidence for the involvement of dynein we would rather not bring this issue up in the discussion and further work that is beyond this study will be required to establish whether dynein is involved in vaccinia transport.

3. As the expression of knock/in TagGFP2-KIF5B was low compared to endogenous KIF5B expression, it is likely that this lead to a considerable underestimation of the number of IMV or IEV associated kinesins. Any idea why the expression of TagGFP2-KIF5B is so weak? As I understood, the authors did select for cells with biallelic expression. Please explain and modify accordingly.

We have no idea why the expression of TagGFP2-KIF5B is so low in the knockin cells. The same was also true for other isolated knockin clones (homozygous and heterozygous). We also found that tagging KIF5B at the C- terminus also resulted in low level of expression in stable cell lines generated with lentiviruses.

The reviewers question promoted us to re-examine the relative levels of KIF5B associated with IMV and IEV in parental and TagGFP2-KIF5B knockin cells (Fig. 7H). We found that IMV and IEV recruited 2.14 and 2.78 fold more kinesin in the parental cells compared to the knockin line (Fig. 7H). We believe this difference reflects the lower expression levels of TagGFP2-KIF5B compared to the untagged KIF5B in the parental cells. Importantly, this analysis clearly indicated that we were underestimating the number kinesin-1 motors on IMV and IEV. The ratio of the corrected values (139 for IMV and 320 for IEV) is now more similar to the fold difference in Fig. 4B (2.30 vs 2.66).

Minor comments:

1. Please add a few sentences explaining the concept of using the nanocages.

We have provided more details when they are first introduced in the results and original reference form the Baker lab.

2. Some abbreviations are not defined: e.g., GMPCPP, BME, PEG, PLL, and KPDG. Definitions provided

© 2022. Published by The Company of Biologists under the terms of the Creative Commons Attribution License (https://creativecommons.org/licenses/by/4.0/).

3. Page 9, line 16: "long" appears to be superfluous - removed

4. Page 10, line 6: missing bracket; - corrected

5. Page 13, last paragraph, line 3: 1 % P/S: remove empty space; - corrected

6. Page 14, lines 4-6: check syntax; - corrected

7. Page 14, line 21: do not capitalize "Glucose". - corrected

8. Page 14, line 27: do not capitalize "Units" - corrected

9. Page 14, line 32: change "MgCl2" to have 2 as a subscript. - corrected

---

Second decision letter

MS ID#: JOCES/2022/260175

MS TITLE: Kinesin-1 transports morphologically distinct intracellular virions during vaccinia infection

AUTHORS: Amadeus Xu, Angika Basant, Sibylle Schleich, Timothy P Newsome, and Michael Way
ARTICLE TYPE: Research Article

I am happy to tell you that your manuscript has been accepted for publication in Journal of Cell Science, pending standard ethics checks.

Reviewer 1

Advance summary and potential significance to field

The authors have done a nice job in addressing my comments and I would suggest acceptance.

Comments for the author

The paper is fine by me now and in my opinion does not require additional experiments.

Reviewer 2

Advance summary and potential significance to field

The manuscript advances our knowledge of how vaccina virus co-opts the microtubule-based trafficking machinery to cause infection

Comments for the author

I am satisfied with the reviewers modifications and responses.

© 2022. Published by The Company of Biologists under the terms of the Creative Commons Attribution License (https://creativecommons.org/licenses/by/4.0/).
